# The Future Is Now: Unraveling the Expanding Potential of Human (Necro)Microbiome in Forensic Investigations

**DOI:** 10.3390/microorganisms11102509

**Published:** 2023-10-07

**Authors:** Ana Cláudia-Ferreira, Daniel José Barbosa, Veroniek Saegeman, Amparo Fernández-Rodríguez, Ricardo Jorge Dinis-Oliveira, Ana R. Freitas, on behalf of the ESCMID Study Group of Forensic and Post-Mortem Microbiology (ESGFOR)

**Affiliations:** 1 1H-TOXRUN, One Health Toxicology Research Unit, University Institute of Health Sciences (IUCS), CESPU, CRL, 4585-116 Gandra, Portugal; a32336@alunos.cespu.pt (A.C.-F.); ricardo.dinis@iucs.cespu.pt (R.J.D.-O.); 2 Instituto de Investigação e Inovação em Saúde (i3S), Universidade do Porto, 4200-135 Porto, Portugal; 3 Department of Infection Control and Prevention, University Hospitals Leuven, 3000 Leuven, Belgium; veroniek.saegeman@uzleuven.be; 4 Microbiology Laboratory, Biology Service, Institute of Toxicology and Forensic Sciences, 28232 Madrid, Spain; amparo.fernandezrodriguez@justicia.es; 5 Department of Public Health and Forensic Sciences, and Medical Education, Faculty of Medicine, University of Porto, 4200-319 Porto, Portugal; 6 UCIBIO—Applied Molecular Biosciences Unit, Laboratory of Toxicology, Department of Biological Sciences, Faculty of Pharmacy, University of Porto, 4050-313 Porto, Portugal; 7 Associate Laboratory i4HB—Institute for Health and Bioeconomy, Faculty of Pharmacy, University of Porto, 4050-313 Porto, Portugal; 8 UCIBIO—Applied Molecular Biosciences Unit, Laboratory of Microbiology, Department of Biological Sciences, Faculty of Pharmacy, University of Porto, 4050-313 Porto, Portugal

**Keywords:** microbiome, necrobiome, thanatomicrobiome, decomposition, bacteria, *postmortem*

## Abstract

The relevance of *postmortem* microbiological examinations has been controversial for decades, but the boom in advanced sequencing techniques over the last decade is increasingly demonstrating their usefulness, namely for the estimation of the *postmortem* interval. This comprehensive review aims to present the current knowledge about the human *postmortem* microbiome (the necrobiome), highlighting the main factors influencing this complex process and discussing the principal applications in the field of forensic sciences. Several limitations still hindering the implementation of forensic microbiology, such as small-scale studies, the lack of a universal/harmonized workflow for DNA extraction and sequencing technology, variability in the human microbiome, and limited access to human cadavers, are discussed. Future research in the field should focus on identifying stable biomarkers within the dominant Bacillota and Pseudomonadota phyla, which are prevalent during *postmortem* periods and for which standardization, method consolidation, and establishment of a forensic microbial bank are crucial for consistency and comparability. Given the complexity of identifying unique *postmortem* microbial signatures for robust databases, a promising future approach may involve deepening our understanding of specific bacterial species/strains that can serve as reliable *postmortem* interval indicators during the process of body decomposition. Microorganisms might have the potential to complement routine forensic tests in judicial processes, requiring robust investigations and machine-learning models to bridge knowledge gaps and adhere to Locard’s principle of trace evidence.

## 1. Introduction

The beginning of microbiology occurred in the late 17th century when van Leeuwenhoek performed the first microscopic observations of bacteria (“little animals”). However, it was only affirmed as a distinct science almost 200 years later, during the middle 1800s, when Louis Pasteur demonstrated that microorganisms were not spontaneously generated [1]. After the global recognition that microorganisms were indeed responsible for human and animal diseases, they have been used as physical evidence along with the beginning of forensic science at the end of the century XIX when the presence of a given infectious agent could be attributed to a specific infection with subsequent death [2]. Since then, microorganisms have been mostly used to associate humans/animals with diseases, fomites, or locations [3,4]. But it was only after the anthrax bioterrorist attack on 11 September 2001, in the USA, that the forensic value of microbiology became a reality with microbiologists being able to apply their experimental results, as trace evidence, for a forensic investigation (in this case, to attribute the source of bacterial spores) [5]. Indeed, the once limited vision of using microorganisms for forensic investigations has gradually changed, running parallel to an unprecedented era of technological sequencing advances since the 2000s—this transformation showed that microorganisms can also serve as temporal evidence once they quickly adapt to environmental changes [1]. Even in the presence of variable conditions (e.g., temperature, oxygen availability, moisture, pH, light), those changes seem temporally predictable, allowing the establishment of a timeline that could be useful in providing information on cadaveric decomposition. One could say that a dead body is a perfect cocktail for several microorganisms such as the chemoorganotrophic bacteria that consume organic compounds (e.g., decomposing remains) to generate energy [6]. Also called decomposers, different bacterial species represent the bulk of microorganisms associated with decomposing remains and trace evidence, and, as microbial signatures are unique, they can be associated with specific hosts or habitats [7].

The development of technologies associated with molecular biology and the large-scale sequencing of microorganisms in recent years, accompanied by advances in bioinformatics and the availability of well-annotated genes/genomes, catapulted forensic microbiology as an emerging discipline with the possibility of multiple applications, namely: (i) providing information on cadaveric decomposition, causes of death and *postmortem* interval calculation; (ii) facilitating the investigation of a bioterrorist attack, biocrimes, outbreaks or product authenticity; and (iii) supporting in crimes of violence, sexual abuse, medical neglect and agri-environmental contamination [7,8].

With the human microbiome playing a key role in the decomposition of *postmortem* tissues, the estimation of the *postmortem* interval (PMI), which refers to the time elapsed since death, is one of the most promising applications of the emerging forensic microbiology area [9]. PMI calculation is one of the main points of investigation of forensic expertise, being, however, influenced by several biotic and abiotic factors that preclude reliable conclusions, at least for now. Traditional methods for estimating PMI calculations commonly rely on taphonomic processes or the assessment of decomposition stages, but as the cadaver undergoes degradation, these approaches become less reliable and challenging to implement effectively [10,11,12]. While different factors that affect the speed of body decomposition (e.g., temperature, moisture, oxygen, etc.) have been extensively studied, the key role of microorganisms in *postmortem* changes has been only recognized and started to be explored more recently [13]. While we witness the continuous improvement of next-generation sequencing methodologies, the occurrence and abundance of particular microbial communities may be at least a complementary tool to obtain more accurate PMI estimations and other forensic investigations in order to be used in medical-legal contexts.

This review aims to document the growing knowledge of the human microbiome composition (both *antemortem* and *postmortem*), its related concepts, and the potential applications in diverse forensic scenarios, with a special focus on the body decomposition process and PMI calculations. The constraints and challenges associated with the different forensic applications and future perspectives on the topic are also discussed.

## 2. Methods

We conducted a narrative review of the literature employing the PubMed and Google Scholar databases concluding our searches on 20 July 2023. We used a combination of key terms, including “microbiome”, “necrobiome”, “thanatomicrobiome”, “decomposition”, “*postmortem*”, “bacteria”, and “forensics”. We meticulously analyzed the titles and abstracts of all articles available in English, without imposing restrictions on publication dates. This process led us to select 97 original articles, 52 review articles, 13 book chapters, 1 editorial, and 1 practical guideline published since 2001 for further full-text review. Exclusion criteria were applied to records lacking full-text access, not written in English, classified as commentaries, conference abstracts, or posters. Articles meeting our criteria of interest were retrieved and thoroughly reviewed, and pertinent data were extracted for inclusion in our study.

## 3. Human Microbiota and Microbiome

The human body is inhabited by trillions of diverse symbiotic microbial cells, including bacteria, archaea, fungi, protists, and viruses. Estimated numbers roughly point to a similar number of microbial and human cells (1:1) with microbial cells including a high number of unique bacterial strains at a given time during life [14]. The colonization of our body is a continuous process, starting in the early neonatal state and succeeding throughout life intimately affected by factors such as ethnic/racial background, diet, lifestyle, host genetics, environment, antibiotic usage, and immunity status, among others [15,16]. The term microbiome describes the entire “ecological community of commensal, symbiotic and pathogenic microorganisms that share our body space” and undergoes intra- and interindividual time variations in terms of number and abundance distribution in different body locations [17,18,19,20]. Microbiota and microbiome terms have been commonly used indistinguishably, though they currently have clearly different meanings. While microbiota refers to the assembly of all living microorganisms (bacteria, archaea, fungi, protists, and viruses) encountered in a given habitat/environment, microbiome refers to the broad collection of all microbial structures, genomes, genetic elements, and metabolites carried by the present microorganisms and embedded within the environmental conditions of that habitat/environment. This review hereafter considers the term “microbiome” to generally include both the microbiota and their “theatre of activity” (structural elements, metabolites/signal molecules, and the surrounding environmental conditions), as previously suggested by Berg et al. [21].

### 3.1. Core Microbiome

The diversity and abundance of human microbial profiles vary significantly from person to person, but there exists a shared group of microorganisms known as the core microbiome. These microorganisms encompass all functional bacterial and genomic taxa that play a crucial role in the proper functioning of the body. They help maintain the regulation of essential functions related to health, including nutritional acquisition, but which, on the other hand, can also be associated with human diseases due to imbalances within such microbiome [15,20,22]. The development of the core human microbiome is a gradual process that begins at birth and continues throughout the first years of life [23]. Even though continuingly evolving across life is influenced by diverse factors, it remains relatively constant across time and amongst individuals if strong microbiota deviations do not occur. However, the definition of a “core” healthy microbiome is debatable, and more research is needed to define it in terms of common sets of taxa, metabolic modules, or other functions. In any case, it should always be considered in the context of its environment, including body parts (as microbial profiles vary between different body sites, including within the same organ), diet, and geography [24,25].

Human microbiota comprises hundreds of bacterial genera and species mainly clustering into four different phyla: the Bacteroidota (former Bacteroidetes), the Bacillota (former Firmicutes), the Actinomycetota (former Actinobacteria), and the Pseudomonadota (former Proteobacteria) [26]. The greatest abundance and diversity of microorganisms are in the gut, which has been the organ most deeply studied regarding microbiome research [27]. The gut microbiome protects against pathogens, provides nutrients and energy, is in constant communication with the immune system, and is involved in many chemical reactions (e.g., drug’s metabolism) having complex effects on human metabolic pathways with implications in several diseases (e.g., inflammatory bowel disease, autoimmune disorders, obesity). Within the gastrointestinal tract, the stomach, duodenum, and ileum have low microbial densities, whereas the jejunum, caecum, and colon are densely populated. The human intestine microbial communities are dominated by the phyla Bacteroidota (e.g., *Bacteroides fragilis*) and Bacillota (e.g., *Lactobacillus* spp., *Faecalibacterium* spp.), with smaller amounts of Pseudomonadota (e.g., *Escherichia* spp.) and Actinomycetota (e.g., *Bifidobacterium* spp.) [28]. This composition is quite unique to each person and remains relatively stable, though suffering some evolution with age and environmental factors, after the first 3–4 years of human life [14]. The beginning of gut colonization is mainly due to facultative anaerobic bacteria (e.g., *Escherichia coli*, *Enterococcus* spp., *Streptococcus* spp., *Staphylococcus* spp.) that, by consuming oxygen, gradually create an anaerobic atmosphere during infancy ready to be colonized with anaerobic bacteria (e.g., *Bacteroides*, *Bifidobacterium*, *Clostridium* spp.) from breast milk [26]. As an example of the complexity and variety of factors when analyzing the human microbiome, the infant diet greatly influences the evolving microbiota. It is known that breast-milk-fed infants have a higher abundance of *Bifidobacterium* and *Bacteroides* compared to those fed formula, who maintain facultative anaerobes (*Enterobacteriaceae*) for longer periods of time [29]. Diet is one of the main factors affecting gut microbiome: for example, in high-fat diets, populations of Bacteroidota and Actinomycetota are often found, while in fiber-based diets the microbiome composition mainly includes Bacillota and Pseudomonadota. Animal-based diets translate into a microbiota with decreased Bacillota (*Roseburia* spp., *Eubacterium rectale* and *Ruminococcus bromii*), and herbal diet shows a great abundance of *Bacteroides* spp. and *Bilophila* spp. [12].

Mouth seems to be the second most diverse habitat of the human body, mainly carrying bacteria but also viruses, fungi, protozoa, and archaea, and gets established in the first year of life. The oral microbiome also maintains a relatively unique composition in each person, although affected at different sites in the mouth (e.g., teeth, tongue, gingiva, saliva). It counts with more than 1000 bacterial species representative of more than 10 phyla (mainly Bacillota, Bacteroidota, Pseudomonadota, Actinomycetota, Spirochaetota, Fusobacteriota) with *Streptococcus* spp., *Fusobacterium* spp., *Lactobacillus* spp., *Actinomyces* spp., *Veilonella* spp. and *Neisseria* spp. among the most abundant genera [30,31]. Saliva, as a sample of the mouth cavity, is particularly important in cases of bite marks, sexual assault, and child abuse. The salivary microbiome is particularly distinct from other sites in the body, but similar among individuals, being, therefore, an indicator of great relevance for PMI estimation. There are approximately 500 million bacterial cells per mL of saliva and this microbiome is highly influenced by individual factors, such as personal oral hygiene and smoking. Saliva naturally contains antimicrobial factors that are no longer produced after death, thus contributing to the *postmortem* microbial invasion in the oral cavity [11]. Gram-positive oral bacteria (e.g., *Streptococcus salivarius*) seem to be robust markers for excessively degraded saliva samples due to their high resistance to degrading factors [32].

The skin is a remarkable example showing great variations in microbiome composition across different skin anatomy sites, according to variable physical and chemical features (lipid content, pH, sweat, and sebum secretion) besides inter-individual variability (e.g., more extensive bacterial diversity in women due to hormonal differences) [33]. The most represented genera in the skin have been generally identified as *Corynebacteria* (Actinomycetota), cutaneous propionibacteria (Actinomycetota), and staphylococci (Bacillota) [34]. For example, the sebaceous glands of the face, scalp, chest, and back are those where large amounts of oily sebum are produced and the preferred to the lipophilic anaerobe *Cutibacterium* (former *Propionibacterium*) *acnes* proliferate. In forensic analyses, *Cutibacterium acnes* can be particularly important since its presence is highly specific and an indication that the sample taken corresponds to a skin sample [20,22]. *Staphylococcus* spp. and *Corynebacterium* spp. are the most common in moist areas, whereas dry areas are more enriched with Pseudomonadota [26]. Bacillota are abundant in more juvenile hands, while *Cutibacterium* is commonly found in adults. This microbiome makes up the barrier between the body and the environment, showing a dynamic flux due to constant exposure to environmental conditions [12,35,36]. A continuing and dynamic flow of microbiota transfer between the skin and surfaces/objects in close contact remaining in the body for an extended period [35,36] exists, but whether this unique microbial fingerprint left behind by skin shedding can be used as trace evidence remains to be validated [36].

The original dogma that the urine is sterile has been knocked down in the last decade and contrary to what was previously thought and to what some articles still describe, a protective urinary or bladder microbiome exists [37]. Although still in its infancy in comparison to other body tracts, studies have described that a healthy female urinary microbiome is highly diverse within and between individuals and dominated by specific family/genera (e.g., *Lactobacillaceae*, *Gardnerella*, *Corynebacterium*) or a mixed community without a prevalent genus (e.g., *Staphylococcus*, *Corynebacterium*, and *Prevotella*) [38]. As such, this must also be taken into account in forensic investigations.

The vaginal microbiome is largely dominated by bacteria having an important role in women’s health and that of their newborns. The healthy vaginal microbiome is dominated by hundreds of bacterial species belonging to Bacillota (e.g., *Lactobacillaceae*, *Streptococcaceae*), Pseudomonadota (e.g., *Enterobacteriaceae*), Actinomycetota (e.g., *Corynebacteriaceae*), and Bacteroidota (e.g., *Prevotellaceae*). A high abundance of *Lactobacillus* species able to produce antimicrobial molecules and lactic acid that maintain an acidic environment against invading pathogens is well recognized [39]. Although relatively stable, the vaginal microbiome varies according to individual characteristics, such as health status, ethnicity, sexual habits, contraceptive use, and pregnancy. The male genital tract has not been studied so extensively compared to the female one. Available studies demonstrate that sperm has a low biomass with high contamination whereas semen has a specific microbiome in healthy fertile individuals, either *Lactobacillus*- or *Prevotella*-enriched or polymicrobial, possibly including members of Actinomycetota (*Corynebacterium*), Bacteroides (*Prevotella*), Bacillota (e.g., *Lactobacillus*, *Streptococcus*, *Staphylococcus*), and Pseudomonadota (*Haemophilus*, *Burkholderia*) [40]. The presence of possible pathogenic bacteria, such as *Ureaplasma urealyticum*, *Mycoplasma hominis*, and *Prevotella* spp. may be related to low semen quality in cases where no spermatozoids are observed [20].

Blood is a fluid commonly found at the place of death, which may originate from distinct places in the body, including menstrual blood, venous blood, nasal blood, and blood from the epithelium of the skin. Blood has been traditionally considered sterile in healthy individuals, just as urine; however, 16S rRNA sequencing studies allowed the distinction of four types of blood. Menstrual blood presents large amounts of species of *Lactobacillus*, nasal blood is affected by the nasal breath that can dilute microorganisms, and blood from the skin epithelium contains the same microorganisms that compose the cutaneous microbiome [41]. Venous blood can present low amounts of bacteria and nonspecific products corresponding to the proteins of the human host. Indeed, human blood is traditionally considered sterile, but the existence of a blood microbiome has become a matter of debate in recent years. A very recent study, which is the most robust to date comprising 9770 healthy individuals, did not support the hypothesis of a core microbiome endogenous to human blood, but instead showed there is a transient translocation of commensal microorganisms from other body sites [42].

Hair is often collected at death scenes for forensic investigations (scalp hair) or sexual assault cases (pubic hair) [43,44]. It has been suggested that the microbiome of pubic hair is more stable and less affected by environmental bacteria. As cohabiting and sexually active couples interchange microbiomes, changes in pubic hair patterns may have an impact on the vaginal microbiome. The presence of *Lactobacillus* sp., characteristic of vaginal samples, is very useful for making this distinction, while *Corynebacterium* is often more commonly found in males. Still, there is not a clear distinction in the microbiota that allows to differentiate male from female, and the length of hair may introduce variations in the microbial communities [20,45].

It is now widely accepted that there is considerable inter-individual variability in the composition of the human microbiome (the often called “personal microbiome”) and it is increasingly possible to establish new biomarkers of disease [14]. There is also a considerable inter-individual fluctuation in the stability of the human gut, tongue, forehead, and palm microbiome so including temporal variability throughout life is a relevant factor to add to the microbiome composition [17]. Notably, and despite the inevitable inter-individual variations, the composition of the human gut microbiome can be distinguished from the communities of other niches such as soil and water [9]. A deep understanding of composition and factors influencing the human healthy microbiome is useful to apply in the investigations related to the thanatomicrobiome and recent years have been fruitful in this respect. Among all microorganisms from the human microbiome, bacteria are, due to their diversity and primary role in the decay process, most relevantly associated with the forensic context [46,47]. Therefore, this review will focus on bacterial communities.

### 3.2. The Necrobiome: Thanatomicrobiome and Epinecrotic Communities

The necrobiome concept intends to reflect all organisms (not only microorganisms but also arthropods and vertebrates) and their genes that interact with decomposing remains (carrion) of heterotrophic biomass. Originally focused on vertebrate carrion, Benbow et al. recently suggested that the term necrobiome should be extended to include microorganisms and any form of necromass such as leaves or wood, for example [48]. It has also been recently demonstrated that the interactions between microorganisms and necrophagous arthropods that colonize decomposing vertebrate carrion affect the rate and timing of decomposition [49]. Thus, the increasing knowledge about this interactive microorganism-arthropod network may support the evolution of forensic sciences in the future.

The microbial communities of the human necrobiome, or *postmortem* microbiome, have been allocated into two body parts: (i) the internal communities have been defined as the thanatomicrobiome (the microbiome of “death”) or the microorganisms found in blood, fluids, and internal organs (e.g., brain, heart, liver, lungs, spleen) upon death; and (ii) the external or epinecrotic communities are found on the surfaces or external body surfaces of decomposing cadavers including inside superficial epithelial tissues, the mouth, ears, eyes, or distal orifices of the digestive tract. The latter ones are easier for collection (noninvasive) but are also more affected by abiotic (e.g., humidity, temperature, and pH) or biotic (e.g., gases, insects, and scavenger activities) factors [11,12,50,51,52]. Expectedly sterile in healthy humans, the internal organs start being invaded by microorganisms after death, which follow a microbial succession in and around the cadaver, greatly influencing body decomposition. The term “thanatomicrobiome” was only introduced in 2014 by Can et al. [53] to avoid confusion with microbiomes encompassing insects, arthropods, or other large organisms that degrade corpses. The knowledge about the thanatomicrobiome composition along the decay process, which is more stable and less biased, may therefore be useful in different forensic contexts [51,54].

#### 3.2.1. Factors Triggering Microbial Invasion after Death

After death, human cells undergo hypoxia, which triggers the activation of autolytic enzymes leading to the degradation of cellular organelles and subsequently breaks down components such as proteins, carbohydrates, and lipids. As a result, an environment rich in nutrients (e.g., nitrogen, carbon, phosphorus, water) propitious for microbial proliferation is created [53]. With the environment getting more hypoxic along the decay process, the degradation shifts to anaerobic fermentation with the release of different gases (e.g., H_2_S, CO_2_, methane, ammonia, sulfur dioxide), and the accumulation of acids (lactic/formic) initiates the *postmortem* fall in pH within the early *postmortem* period [55]. As soon as the immune system decays, microorganisms originating from inside (the healthy microbiota in most cases or from infections if that’s the case) or outside (microorganisms or even flies from the local environment) can enter the body, including into usually sterile organs, and increase the overall microbial load [56]. Temperature and anaerobic conditions have been pointed out as the main factors driving this decomposition process [57].

In addition to the factors aforementioned, changes that occur in the *postmortem* microbiome, namely in organs and fluids usually sterile, are influenced by the existence of migration phenomena, such as the agonal spread of microorganisms (*perimortem*), *postmortem* translocation or contamination (less probable with strict precautions during sampling) [50,57,58]. Genuine positives can also occur in the case of a *premortem* bacterial infection, but these usually generate a pure culture growth instead of mixed growth populations except in some polymicrobial infections such as most peritonitis involving intestinal microbiota. The damage to the mucosal surfaces’ integrity by the agonal spread or invasion of microorganisms into the bloodstream, subsequent to the ischemia/hypoxia, is controversial due to the difficulty of proving its existence during death or when systemic circulation is artificially maintained by resuscitation attempts. Much more evidence is available regarding *postmortem* translocation, which is marked by the entry of intestinal and mucosal bacteria into the circulatory and lymphatic systems, progressing later to other organs due to the decay of the immune system at the time of death. This can be avoided, or at least diminished, if corpses are kept at 4 °C as soon as they are found and preferably within 24 h after death [50,52].

During a person’s lifetime, the translocation of different bacterial types (aerobic and anaerobic) can occur. This translocation is associated with the migration of viable bacteria or bacterial fragments from the gut to the mesenteric lymph nodes where bacteria are typically eliminated, preventing their further dissemination. However, in certain cases, translocated bacteria can bypass this elimination process and reach the systemic circulation ultimately leading to sepsis [59]. Some bacteria seem to have a particular potential for translocation (e.g., *Enterobacteriaceae*, *Enterococcus*, *Clostridia*), which can also happen in healthy individuals without negative outcomes if the immune system is able to eliminate them. Different damages/conditions may account for this phenomenon both during life or after death [60]: (i) intestinal mucosa alterations, such as changes in mucus composition or secretion are commonly associated with specific conditions like bowel inflammatory diseases including Crohn’s disease; (ii) modification of the intestinal microbiota (hemorrhagic shock or antibiotherapy); and/or (iii) immunodeficiency (e.g., alterations of T lymphocytes signal, a decrease in IgA). *Postmortem* translocation may be facilitated by an increased permeability of the gut wall due to a long agonal phase, the absence of blood supply responsible for ischemia, the absence of mucus secretion, or even a medical history, such as an intestinal bowel disease. The detection of bacteria in organs, such as the brain, liver, spleen, and heart that are theoretically sterile unless there is a true infection, is an indicator of bacterial migration [50,52].

#### 3.2.2. Body Decomposition and Microbial Succession after Death

Cadaveric decomposition is a dynamic ecological system that undergoes continuous evolution, primarily driven by microbial and necrophagous activities. This process can be broadly divided into five main stages, each with distinct characteristics [fresh decay (autolysis), bloat (putrefaction), active decay (black putrefaction), advanced decay (butyric putrefaction) and skeletonization or dry (diagenesis)] that provide us with essential information for PMI knowledge, location, circumstances, and cause of death [36,61,62,63,64]. The rate and pattern of decomposition are an irreversible mosaic system of physical and biochemical changes associated with biotic factors, such as pathologies and personal individualities, intrinsic bacteria, and abiotic factors [10,11,12,36,46]. Bacteria occupy several internal and external sites of the body and derive not only from the inside of the cadaver but also from the vertebrate scavengers, arthropods, and soil where it is located, thus having a redoubled influence on the decomposition process. Different factors and scenarios (e.g., weather conditions, season, *antemortem* individuality, corpse *postmortem* manipulation, etc.) combine to generate unique scenarios of decomposition. [36,46,62,63,64,65]. Notably, it has been shown that the interactions between microbes and the scavenging arthropods that colonize the carcass of decomposing vertebrates affect the rate and time of decomposition, and can influence the *postmortem* microbiome. As so, the growing knowledge about this microbe-arthropod interactive network can support the evolution of forensic science in the future [66].

Initial insights into the microbial communities associated with decomposition were made in nonhuman models in the 1980s [67]. Though the first studies assessing bacterial gene markers, so using non-culturable methods, in human cadavers only started in 2013 [46]. Hyde et al. [67] described that rectal/stool samples contained the most diverse bacterial community, while the stomach contained the least diverse bacterial community (dominated by the acid-tolerant *Morganella*, a Pseudomonadota). In general, available studies provide a description of microbial taxa and characterize decomposition patterns during the decomposition process, because it is hard to test different experimental manipulations that can be compared to control conditions. Still, such pattern-oriented data generate relevant information to conduct future studies.

Although still in its infancy, if compared to the extensive knowledge made on forensic entomology, the number of studies addressing microbial composition during cadaver decomposition is exponentially increasing. Such boom is greatly attributed to the modern culture-independent methods providing more complete and robust data, and because most bacteria from the decomposing community are non-cultivable by traditional culture techniques [67]. The ongoing amount of data that is daily generated is currently higher than ever and strongly dependent on the sequence technology and model used (explored in Section 5).

Decomposition starts quickly after death with the activity of microorganisms, pancreatic enzymes, and gastric acids hypothetically in a certain order, such as larynx and trachea, stomach, intestine, spleen, liver, pancreas, pregnant uterus, heart, lungs, kidneys, urinary bladder, while the skin, muscles, tendons, bones and nulligravid uterus are the last elements to degrade [68,69]. During fresh decay, bacteria inside the body initiate the digestion of surrounding tissues through cell autolysis or self-digestion, resulting in the release of nutrients and macromolecules that are then metabolized by resident microorganisms, especially those from the gut, facilitating the decomposition process. Moreover, a marked shift from aerobic (requiring oxygen to grow) to anaerobic (not requiring oxygen) species occurs, with the latter fermenting sugar in body tissues and producing gaseous by-products (e.g., methane, ammonia). These fermentative processes, characterized by the accumulation of gases and the distension of the body, trigger the beginning of the swelling, inflating, or bloat phase, especially in the abdomen, eventually forcing fluids out of the body (purge). Such purging events mark the transition from early to late decomposition and are not necessarily uniform among the different body sites. This enables the breakdown of proteins, wet tissue decomposition, and the release of byproducts leading to discoloration and the strong odor typically associated with decaying bodies. The advanced decay stage is marked by the breakdown of fats and the production of volatile fatty acids, contributing to the unique smell of decomposition. Putrefaction is accelerated by vertebrate scavengers or necrophilous arthropods consuming the soft tissues and gradually leading to the dry stages of decomposition (the carcass is reduced to bone, cartilage, and any unconsumed tissue or hair) [3,20]. Microorganisms usually start spreading from the gut, digest the intestines, and then the surrounding tissues using the chemicals leaked from the damaged cells. Afterward, they invade the digestive system and lymph nodes, spreading first to the liver and spleen, followed by the heart and brain [50,52].

Different studies showed an increase in bacterial richness and a decrease in diversity from the early to late decomposition stages (Table 1) [9,70]. These studies have also consistently demonstrated an increase in Pseudomonadota and a decrease in Actinomycetota and Bacteroidota, with particular relevance in the rectum [71,72]. Additionally, another study has reported a negative linear relationship between the overall phylum and family taxa with PMI. For instance, Moraxellaceae showed an increase on the day of death, while Aerococcaceae and Enterobacteriaceae were no longer detectable after the fifth day *postmortem* [3]. This shift in the cadaveric microbial composition from early to late stages happens during bloating, which is often used as a marker for such transition [46,70]. When the cadaver enters the fresh stage, the microbial community associated is, at the level of phylum, Bacillota and Actinomycetota, Lactobacillaceae, Staphylococcaceae, Gemellaceae, Carnobacteriaceae, Aerococcaceae, Veillonellaceae, Streptococcaceae, Campylobacteraceae, Micrococcaceae, Bifidobacteriaceae, Actinomycetaceae, and Corynebacteriaceae in the level of family [73]. Entering the bloat phase, a phase of fermentation and proteolysis, the abundant phyla are Bacillota and Tenericutes, having the class of Clostridiales, families of Peptostreptococcaceae, Bacteroidaceae, Enteranococcaceae, and the genus *Ignatzschineria* [73]. In the active decay, there is a change from the aerobic *Staphylococcus* and Enterobacterales to an anaerobic environment where bacteria able to survive in these conditions dominate, such as Pseudomonadota, Bacteroidota, *Clostridium*, *Bacteroides*, *Enterococcus*, *Proteus* and many others from the surrounding environment [74,75,76,77,78]. When the corpse reaches the advanced stage, there are changes in the microbial community in the cadaver, frequently encountering Bacillota, Gammaproteobacteria, Pseudomonadaceae, Alcaligenaceae, and Planococcaceae [73]. When the remains are practically skeletal, the bacterial communities associated with them are close to the intestinal communities, being *Bacillota* and *Bacteroidota*, as well as *Acidobacteria, Actinomycetota, Pseudomonadota, Planococcaceae*, *Enterococcus*, *Vagococcus*, *Clostridium*, *Corynebacterium*, *Proteus*, and *Acinetobacter* dominant [3,74,75,79]. In this dry stage, bacterial communities are similar to soil communities [12,75]. After 420 days of burial, the succession of bacterial communities in the soil is complete [12,35,65].

Importantly, some studies addressed statistically significant time-, organ-, and sex-dependent changes, a variability that is also seen in current microbiome studies analyzing healthy live samples [54]. Some of the largest efforts to assess microbial diversity of internal components of the human thanatomicrobiome showed that Bacillota (former Firmicutes) could be potential biomarkers [54,80]. Still, bacteria belonging to this phylum may face a decrease instead of an increase in particular body sites such as the mouth and rectum. Adding to the confusion, contrasting data are also available: while Hyde et al. [79] described an increased abundance of Bacillota in the mouth over time, Guo et al. [11] reported a decrease. Besides the inter-individual variability and the possible differences in environmental and biological conditions (weather, clothing…), they used different timepoints and DNA extraction methods. In any case, different studies point to similar bacterial groups as being key *postmortem* taxa involved in decomposing cadavers, which mainly belong to Gammaproteobacteria, *Lactobacillaceae*, and *Clostridiaceae* [3,46,53,80,81].

**Table 1:** Literature evidence on microbial signatures before death and along the body decomposition process.

Body Site	After Death		
	Overall Changes ^a^	Model Used and Timepoints	References
**Body**	Richness ↑ Diversity↓	Human (*n* = 6); 0–20 d; Human (*n* = 4) 0–30 d	[9,70]
	Richness ↓ (except in the rectum) Actinomycetota and Bacteroidota ↓ Pseudomonadota ↑	Human (*n* = 188); <48 h/>49 h (2 timepoints)	[72]
	*S. aureus* KUB7 5–7 d ↑and then decrease until no detection at 30 d *S. aureus* highest concentrations by culture on 5 d for surface sterilized mice *S. aureus* highest concentrations by culture on 7 d for non-surface sterilized mice	Mice (*n* = 90); 1 h–60 d (9 timepoints)	[82]
	Dominance of *Clostridium* spp. in internal *postmortem* communities; **Bacillota suggested as a stable biomarker** Female: high abundance of *Pseudomonas* and Clostridiales Male: high abundance of Clostridiales and *Streptococcus*; exclusive presence of *Rothia* *Clostridium* and *Prevotella* species as predictive of different periods of decomposition	Human (*n* = 27); 3.5–240 h (66 timepoints)	[54]
	Richness ↓ Bacteroidaceae and Moraxellaceae were good indicators in the initial sampling; Bacillaceae/Clostridiales were significant after 5 d Pseudomonadota was dominant followed by Bacillota Pseudomonadota ↓ over time until 5 d Bacillota ↑ over time Moraxellaceae ↑ 0 d Aerococcaceae, Enterobacteriaceae ↑ 3 d and no presence after 5 d Planococcaceae, Clostridiales, Clostridiaceae—dominant at 5 d	Swine (*n* = 3); 0–5 d (4 timepoints)	[3]
	*Ignatzschineria* and *Wohlfahrtimonas* were common at bloat and purge and until tissues began to dehydrate *Acinetobacter* were common after dehydration and skeletonization *Ignatzschineria* dominated during the wettest phases and ↓ until skeletonization *Ignatzschineria* was less abundant and less persistent *Wohlfahrtiimonas* associated with myiasis	Human (*n* = 2); 1–20 d (10 timepoints)	[79]
**Skin**	Bacteroidota (Sphingobacteriaceae), Alphaproteobacteria (Brucellaceae, Phyllobacteriaceae, and Hyphomicrobiaceae), and Betaproteobacteria (Alcaligenaceae) ↑ during the advanced decay. Taxa in Rhizobiales were among the most important predictive taxa at each sample site.	Mouse (*n* = 40); 0–48 days (8 timepoints)	[80]
	Dominated by Pseudomonadota at first 2 d ↑ Bacillota, Actinomycetota during the later phases *Pseudomonas and Acinetobacter* were dominant before purging ↑ *Ignatzschineria* after purge and ↓ at dry stage *Clostridium* dominated in the later phases	Human (*n* = 2); 1–20 d (10 timepoints)	[79]
	*Clostridium* ↑ max. at 5 d and 7 d	Mice (*n* = 90); 1 h–60 d (9 timepoints)	[82]
**Blood**	At 5 min, 25% culture-positive to enterococci, lactobacilli, and/or *Bacteroides/Prevotella* spp. At 1 h, bacterial translocation rates were lowest (virtually no bacterial growth) Culture-positive until 30 min, ↓ at 1 h, ↑ to max. at 48 h and 72 h At 72 h, culture-positive for *E. coli* (100%), enterococci (75%) and lactobacilli (62.5%)	Mice; 0–72 h (10 timepoints)	[83]
**Brain**	Dominated by MLE1-12 (*Candidatus Melainabacteria*), Saprospirales and Burkholderiales ↑ Relative abundance in ASVs belonging to the order *Clostridiales* ↓ Relative abundance in ASVs belonging to the order MLE1-12 (not significant)	Human (*n* = 40); 24–432 h	[19]
	Bacteroidota and Pseudomonadota showed different succession patterns At the genus level, *Ochrobactrum* and *Sediminibacterium* were dominant, and ↓ with PMI progression ↑ *Acinetobacter*, *Cupriavidus*, and *Agrobacterium (were dominants)* At the phylum level, **Pseudomonadota was the most prevalent** ↑ *Deinococcota* during 12 h At the order level, Rhizobiales was dominant ↓ Saprospirales, Caulobacterales and Thermales ↑ Burkholderiales and Pseudomonadales during 1 d ↑ *Acinetobacter* at 8 h; ↑ *Cupriavidus* and *Agrobacterium* after 8 h	Mice (*n* = 30); 0:30 h–1 d (5 timepoints)	[84]
**Eyes**	↑ *Streptococcus* early in PMI ranges (<24 h, 25–48 h)	Human (*n* = 188); <48 h/>49 h (timepoints)	[72]
**Oral cavity/Mouth**	↑ Pseudomanodota followed by ↑ Bacillota *Pseudomonas* and Enterococcaceae dominated before purging Planococcaceae dominated after purging and then dropped off as ↑ Clostridium	Human (*n* = 2); 1–20 d (10 timepoints)	[79]
	*Pseudomonas* was detected in pre-bloat but was not in any end-bloat At the end-bloat stage, *Pseudomonas* was replaced by common GI tract bacteria (Clostridia, *Lactobacillus*, etc.) Streptococcus, Prevotella, and Veillonella detected in pre-bloat swab and scrape Pre-bloat swab and end-bloat scrape was predominated by Bacillota Pre-bloat scrape was predominated by Pseudomonadota	Human (*n* = 2); 0–30 d (8 timepoints)	[46]
	**Pseudomonadota showed a positive linear correlation with PMI** **↓ Alpha diversity over decomposition time** **Pseudomanodota and Bacillota were dominant** Pseudomanodota ↓ first and then ↑ Bacillota↑ first and then ↓ Actinomycetota and Bacteroidota ↓ At 0 h, abundance of Pseudomonadota (*Acinetobacter*, *Pseudomonas*, *Phyllobacterium*, *Photobacterium*, *Vibrio*, *Arcobacter*, *Muribacter*) and Actinomycetota (*Propionibacterium*, *Rhodococcus*), Bacillota (*Ruminococcaceae*_UCG-014, *Clostridium* sensu_stricto_1, *Paeniclostridium, Lactobacillus, Christensenelaceae*_R-7_Group), Bacteroidota (*Alistipes*, *Prevotella* _9, *Marinitilum*) and Fusobacteria (*Fusobacterium*, *Psychrilyobacter*). At 24 h, abundance of Bacillota (*Blautia*, *Enterococcus*, *Streptococcus*, *Faecalbacterium*), Pseudomonadota (*Pasteurella*), Bacteroidota (*Bacteroides*), Actinomycetota (*Bifidobacterium*). At 144 h, abundance of Actinomycetota (*Staphylococcus*, *Subdoligranulum*, *Romboutsia*) and Pseudomonadota (*Morganella*, *Escherichia shigella*, *Enterobacter*). At 240 h, abundance of Pseudomonadota (*Citrobacter*, *Proteus*) ↓ Alpha-proteobacteria and Bacteroidia ↑ Gammaproteobacteria Bacilli and Clostridia ↑ first and then ↓ ↑ Enterobacterales, ↑ *Proteus* ↓ *Pasteurellales*, *Bacteroidales* and *Rhizobiales* *Lactobacillales* ↑ first and then ↓ ↓ Pasteurellaceaeae and Phyllobacteriaceae Streptococcaceae, Ruminococcaceae, and Bacteroidaceae ↑ first and then ↓ *Muribacter* and *Phyllobacterium* ↑ first and then ↓	Mice (*n* = 24); 0–240 h (4 timepoints)	[10]
	**Microbial communities were similar in diversity over decomposition time** **↓ Alpha diversity over decomposition time** *Haemophilus parainfluenzae* and *Streptococcus* were most abundant at <24 h and 25–48 h Bacteroidota (e.g., *Prevotella*) during the earlier stages of decomposition *Streptococcus* was a predominant taxon during pre-bloat and during the first 4 d ***Streptococcus* as a potential biomarker during the first 2 d** ***H. parainfluenzae* potential bioindicator <48 h after death**	Human (*n* = 188); <48 h/>49 h (2 timepoints)	[72]
	**Bacillota and Actinomycetota are the predominant phyla in the fresh stage** ***Tenericutes’* presence corresponds to the bloat stage** **Peptostreptococcaceae and Bacteroidaceae were predominant families in the bloat stage** **Bacillota is the predominant phyla in advanced decay** (different community from the fresh stage) The fresh stage was characterized by *Lactobacillaceae*, *Staphylococcaceae*, *Gemellaceae*, *Carnobacteriaceae*, *Aerococcaceae*, *Veillonellaceae*, *Streptococcaceae*, *Campylobacteraceae*, *Micrococcaceae*, *Bifidobacteriaceae*, *Actinomycetaceae* and *Corynebacteriaceae.* Bacillota and Actinomycetota predominant from 1 d to 5 d, but their relative abundances ↓ from 1 d to 5–6 d ↑ Bacillota 6–12 d (Clostridiales and Bacillaceae—representative Bacillota from bloat to advanced decay stages) ↑ *Tenericutes* transiently between 5 d and 7 d, just at the bloat stage ↑ *Ignatzschineria* and Clostridiales in the bloat stage Gammaproteobacteria, Pseudomonadaceae, Alcaligenaceae, and Planococcaceae are predominant families in advanced decay Bacillia nd Clostridia presence in skeletonization/dry stage	Human (*n* = 3); 1–12 d (7–8 timepoints)	[73]
**Buccal Cavity**	↑ Alpha diversity after death At 4 h, Bacillota and Actinomycetota were dominant Bacillota gradually ↓ At 1 d, ↑ Pseudomonadota (predominant phylum) and ↑ Moraxellaceae (predominant family) and gradually ↓ At 2 d, Enterobacteriaceae dramatically ↑ and ↓ at 4 d Xanthomonadaceae gradually ↑ (dominant taxon from 3 d) At 6 d, ↑ Pseudomonadaceae Streptococcaceae and Pasteurellaceae gradually ↓	Rat (*n* = 18); 1–9 d (9 timepoints)	[11]
**Heart**	Dominated by MLE1-12 (*Candidatus Melainabacteria*), Saprospirales and Burkholderiales ↑ Relative abundance in ASVs belonging to the order Burkholderiales ↓ Relative abundance in ASVs belonging to the order MLE1-12 (not significant)	Human (*n* = 40); 24–432 h	[19]
	*S. aureus* remained at 0 until 7 d, ↑ to max. after 14 d ↑ and ↓ to levels near zero at 30 d At 5 h, a sample showed 100% *Escherichia* and others have *Candidatus Arthromitus*, *Parabacteroides*, *Anaerostipes*, and *Dorea* At 7 d, *Clostridium* dominated (72.1%) with *Lactobacillus* and *Peptostreptococcaceae* spp.	Mice (*n* = 63); 1 h–30 d (7 timepoints)	[62]
	Varying numbers of *Clostridium* from 1 h to 24 h, that reached and remained at max. countable limits 5 d to 14 d; *Clostridium* isolates were also recovered at 30 d and 60 d	Mice (*n* = 90); 1 h–60 d (9 timepoints)	[82]
	At the genus level, *Thermus* was more abundant ↓ *Enhydrobacter and Caulobacter, belonging Alphaproteobacteria* and *Methyloversatilis* during 1 d ↑ *Pseudomonas* at 8 h ↑ *Sphingomonas* and *Cupriavidus* to peak values at 12 h At the phylum level, Pseudomonadota and Deinococcota were dominant *perimortem* ↑ Bacillota and ↓ Actinomycetota during 1 d At the order level, *Pseudomonadales*, *Thermales*, and *Burkholderiales* were dominant ↑ *Sphingomonadales* to a peak value at 12 h ↑ *Rhizobiales* during 1 d ↑ *Deinococcales* at 12 h ↓ *Rhodocyclales*, *Rhodospirillales*, and *Caulobacterales* during 1 d	Mice (*n* = 30); 0:30 h–1 d (5 timepoints)	[84]
**Pericardial Fluid**	*Streptococcus* sp. isolates found 5–7 d *Clostridium* sp. isolates found 1–3 d *Clostridium* sp., *Enterobacter* sp., *Bifidobacterium* sp., *Bacteroides* sp. ↑	Human (*n* = 33); 1–7 d (3 timepoints)	[85]
**Lungs**	*S. aureus* at 5 h *postmortem* ↓ to 0, after 5 h ↑↑ to max. at 14 d and ↓ up to 30 d At 5 h PM, contained 100% *Lactobacillus* At 7 d, contained 44% *Clostridium* and 55% *Staphylococcus*	Mice (*n* = 63); 1 h–30 d (7 timepoints)	[62]
	Varying numbers of *Clostridium* from the 1 h to 24 h, that reached and remained at max. countable limits 5 d to 14 d *Clostridium* isolates were also recovered at 30 d and 60 d	Mice (*n* = 90); 1 h–60 d (9 timepoints)	[82]
**Abdominal cavity**	Bacillota (Lactobacilaceae, e.g., *Lactobacillus*) and Bacteroidota (Bacteroidaceae, e.g., *Bacteroides*) ↑ during the bloating stage (6–9 d) Bacillota (Lactobacilaceae, e.g., *Lactobacillus*) and Bacteroidota (Bacteroidaceae, e.g., *Bacteroides*) ↓ after rupture occurs (∼9 d) Rhizobiales (Alphaproteobacteria) in the families Phyllobacteriaceae, Hyphomicrobiaceae, and Brucellaceae (e.g., *Pseudochrobactrum* and *Ochrobactrum*) dominate *Serratia*, *Escherichia*, *Klebsiella*, and *Proteus* become abundant after rupture	Mouse (*n* = 40); 0–48 days (8 timepoints)	[80]
**Gut**	Total bacteria load ↑ Relative abundances ↓ ↓ *Bacteroides* and *Lactobacillus* over time *Bifidobacterium* no significant change over the study	Human (*n* = 6); 0–20 d	[9]
	Enterobacterales and *Escherichia* were detected in the lower GI tract for both pre-bloat and end-bloat *Clostridium* is abundant at the end of the bloat stage	Human (*n* = 2); 0–30 d (8 timepoints)	[46]
	Bacteroidales (*Bacteroides*, *Parabacteroides*) ↓ Clostridiales (*Clostridium*, *Anaerosphaera*) and Gammaproteobacteria, *Ignatzschineria* and *Wohlfahrtiimonas* ↑ Relative abundances and diversity ↓ *Bacteroides*, *Parabacteroides* and *Lactobacillus* ↓	Human (*n* = 4); 0–30 d	[70]
	Total bacterial load ↑ 12 h and 24 h post sacrifice with high levels of enterobacteria and lactobacilli Total bacterial load ↓ 15 and 30 min post sacrifice with ↓ Enterobacteria, enterococci, bifidobacteria, and *Clostridium* spp. Enterobacteria, enterococci, bifidobacteria, and *Clostridium* spp. ↑ to de max. levels from 30 min until the end of the study Varying numbers of *Clostridium* from the 1 h to 24 h, that reached and remained at max. countable limits 5 d to 14 d *Clostridium* isolates were also recovered at 30 d and 60 d	Mice (*n* = 90); 1 h–60 d (9 timepoints)	[82]
	Until 5 h postmortem *Parabacteroides*, *Mucispirillum*, and *Lactobacillus* dominated At 24 h ↓ relative abundance of *Parabacteroides*, disappearance of *Mucispirillum* and ↑ *Lactobacillus* At 7 d ↓ *Lactobacillus* and ↑ *Anaerostipes, Clostridium*, and *Enterococcus* *Staphylococcus aureus*—stable 1–5 h, ↓ at 24 h, ↑ to max. after 7 d and ↓↓ to min. at 14–30 d	Mice (*n* = 63); 1 h–30 d (7 timepoints)	[62]
	***Lactobacillus*, *Dubosiella*, *Enterococcus*, and *Lachnospiraceae*—proposed as significant biomarkers** *Bacillota* (*Lactobacillus reuteri*/*johnsonii*, *Clostridium tetani*, *Enterococcus faecalis*), *Bacteroidota*, *Actinomycetota*- dominant Bacteroidota e Actinomycetota 2 d↑—2 d-4 d↓ *Bacillota bacterium* M10-2—appeared on 2 d and 2 d-4 d↑ *Enterococcus faecalis*—appeared on 2 d and 2 d-10 d↑ *Tenericutes* (bloat phase) *Lactobacillus reuteri* ↑—peak values 7 d and 15 d *Clostridium tetani* E88—appeared on 7 d until 15 d and then ↓ *Lactobacillus johnsonii* ↑ 1 week after death *Helicobacter* ↓ gradually during 15 d *Gordonibacter*, *Bifidobacterium*, *Enterorhabdus*, *Lactococcus*, *Clostridium sensu stricto*, *Anaerosalibacter*, *Enterococcus*, *Dubosiella*, *Lactobacillus*—remained at 15 d	Mice (*n* = 240); 6–10 w (10 timepoints)	[86]
**Colon**	Total bacterial load ↓ between 3 h and 6 h with ↓ lactobacilli and *Bacteroides/Prevotella* spp. ↑ Enterococci between 6 h and 12 h and remain stable until 72 h Lactobacilli ↓ between mice alive and 72 h *Escherichia coli* remained stable at 0 until 72 h *Bacteroides/Prevotella* spp. ↓ 3–12 h	Mice; 0–72 h (10 timepoints)	[83]
	*Bifidobacterium* detected at end-bloat	Human (*n* = 2); 0–30 d (8 timepoints)	[46]
**Ileum**	↑ Distinct in fastly replying aerobic species between 6 h and 24 h Total eubacterial loads ↑ 72 h with max. loads of enterobacteria, enterococci and lactobacilli Enterobacteria ↑ between 3 h and 12 h Enterococci ↑ between 6 h and 24 h Enterobacteriaceae 12 h–72 h↑ Enterococci 24–72 h↑ *Lactobacilli* significantly ↓ until 72 h *Bacteroides/Prevotella* spp. ↑3 h, ↓12 h, ↑72 h *Clostridium coccoides* and *leptum* groups ↑3 h, ↓12 h, ↑72 h Mouse Intestinal *Bacteroides* ↑3 h, ↓12 h, ↑72 h Bifidobacteria ↑6 h, ↓24 h	Mice; 0–72 h (10 timepoints)	[83]
**Rectum**	Taxon richness first ↓ and then ↑ Bacillota, Pseudomonadota, Bacteroidota, and Actinomycetota were found at all the timepoints At the phylum level, Pseudomonadota and Bacillota showed major shifts At the phylum level, bacterial richness ↓ from 0 h to 9 d and ↑ from 9 d to 15 d At the family level, Prevotellaceae, Muribaculaceae, and Lachnospiraceae ↓ at 0 h, 8 h, 16 h, 3 d, 7 d, 15 d At the family level, bacterial richness ↓ from 0 h to 9 d and ↑ from 9 d to 15 d At the genus level, *Lactobacillus* dominated at 1 d and *Enterococcus* from 3 d to 13 d Bacteroidota ↓↓ after death, but ↑ at 3 d and 15 d Actinomycetota relative abundances ↓ at 16 h, 7 d, and 15 d Bacillota and Pseudomonadota peak values at 8 h, 1 d, and 9 d *Helicobacter* was absent at 7 d, 9 d and 15 d ↑ Lactobacillaceae, Enterobacteriaceae, and Enterococcaceae represented the majority from 0 h to 15 d *Enterococcus* and *Vagococcus* relative abundances ↑ at 0 h, 8 h, 3 d, 7 d and 15 d *Proteus* was most abundant at 15 d At the species level, *Enterococcus faecalis* ↓ and *Proteus mirabilis* ↑ after 5 d *Clostridium sporogenes* ↓ abundance before 1 d and *Falsiporphyromonas_endometrii* after 3 d *E. faecalis* and *P. mirabilis* appeared during the whole 15 d	Rat (*n* = 8); alive-15 d (11 timepoints)	[74]
	Bacteroidota and Bacillota were the predominant phyla until 2 d Prevotellaceae was the predominant family until 2 d Pseudomonadota was the most abundant phylum after 2 d Enterobacteriaceae was a predominant family after 2 d	Rat (*n* = 18); 1–9 d (9 timepoints)	[11]
**Feces**	Bacteroidota and Bacillota were the most abundant phyla before purging Pseudomonadota dominated after purging until the drier phases ↑ Bacillota and Actinomycetota in dry phases Clostridiaceae, Bacteroides, and Porphyromonas presented before purging *Corynebacterium* was the most abundant at the dry stage *Ignatzschineria* ↑ to max. after purge and ↓ at the dry stage *Clostridium* became the most abundant at the dry stage Clostridiaceae were the most abundant at the dry stage	Human (*n* = 2); 1–20 d (10 timepoints)	[79]
	Bacillota mainly dominated with very few Bacteroidota detected in a sample Pseudomonadota dominated in another sample *Pseudomonas* was detected in pre-bloat but was not in any end-bloat At the end-bloat stage, *Pseudomonas* was replaced by other GI tract bacteria (Clostridia, *Lactobacillus*, etc.)	Human (*n* = 2); 0–30 d (8 timepoints)	[46]
**Liver**	Sterility up to 5 d After 5 d, *Clostridium* sp., *Streptococcus* sp., *Enterobacter* sp., *Enterococcus* sp., *Escherichia* sp., *Staphylococcus* sp. and *Streptococcus* sp.	Human (*n* = 33); 1–7 d (3 timepoints)	[85]
	Dominated by MLE1-12 (*Candidatus Melainabacteria*), Saprospirales and Burkholderiales ↑ Relative abundance in ASVs belonging to the order *Clostridiales* ↓ Relative abundance in ASVs belonging to the order MLE1-12 (not significant)	Human (*n* = 40); 24–432 h	[19]
	Varying numbers of *Clostridium* from the 1 h to 24 h, that reached and remained at max. countable limits 5 d to 14 d *Clostridium* isolates were also recovered at 30 d and 60 d	Mice (*n* = 90); 1 h–60 d (9 timepoints)	[82]
	At 1 h, bacterial translocation rates were lowest (virtually no bacterial growth) Culture-positive until 30 min, ↓ at 1 h, ↑ to max. at 48 h and 72 h.	Mice; 0–72 h (10 timepoints)	[83]
	At the genus level, *Thermus* and *Cupriavidus* were dominant ↓ *Microbacterium* to zero at 24 h ↑ *Acinetobacter*, *Cupriavidus*, and *Pseudomonas* over decomposition Genera *Paracoccus* and *Cryocola* were detected only at 0:30 h At the phylum level, *Pseudomonadota* and *Deinococcota* were dominant *Actinomycetota*, *Bacillota*, *Bacteroidota*, and *Cyanobacteria* showed relative abundances of > 1% ↓ *Actinomycetota* during 1 d At the order level, *Burkholderiales*, *Pseudomonadales*, and *Thermales* were dominant ↑ Clostridiales during 1 d ↓ *Actinomycetales;* ↓ *Rhodobacterales* during 4 h Comamonadaceae, a family of Betaproteobacteria, was also significantly enriched	Mice (*n* = 30); 0:30 h–1 d (5 timepoints)	[84]
**Spleen**	Varying numbers of *Clostridium* from the 1 h to 24 h, that reached and remained at max. countable limits 5 d to 14 d *Clostridium* isolates were also recovered at 30 d and 60 d	Mice (*n* = 90); 1 h–60 d (9 timepoints)	[82]
	Dominated by MLE1-12 (*Candidatus Melainabacteria*), *Saprospirales* and *Burkholderiales* ↑ Relative abundance in ASVs belonging to the order *Clostridiales* ↓ Relative abundance in ASVs belonging to the order MLE1-12 (not significant)	Human (*n* = 40); 24–432 h	[19]
	At 1 h, bacterial translocation rates were lowest (virtually no bacterial growth) Culture-positive until 30 min, ↓ at 1 h, ↑ to max. at 48 h and 72 h.	Mice; 0–72 h (10 timepoints)	[83]
**Kidney**	*S. aureus* KUB7 detected 1 h post sacrifice; not detected at 3 h, 5 h, 24 h post sacrifice of surface-sterilized mice and detected again 5 d through 14 d Surface sterilized mice—*Clostridium* ↑ max. at 5 d and 7 d and ↓ at 14 d, 30 d, and 60 d Non-surface sterilized mice—*Clostridium* ↑ max. at 7 d and 14 d and ↓ at 30 d and 60 d	Mice (*n* = 90); 1 h–60 d (9 timepoints)	[82]
	At 1 h, bacterial translocation rates were lowest (virtually no bacterial growth) Culture-positive until 30 min, ↓ at 1 h, ↑ to max. at 48 h and 72 h.	Mice; 0–72 h (10 timepoints)	[83]
	At the genus level, *Thermus* was dominant ↑ *Acinetobacter* and *Pseudomonas* during 8 h; ↓ *Methyloversatilis* during 1 d At the phylum level, Pseudomonadota, Deinococcota and Bacillota were dominant ↓ Fusobacteria and Cyanobacteria during 1 day ↑ Pseudomonadota and Actinomycetota At the order level, Pseudomonadales and Thermales were dominant ↓ Streptophyta, Clostridiales, and Rhodocyclales during 1 d ↑ Burkholderiales, Rhizobiales, Bacteroidales and Actinomycetales	Mice (*n* = 30); 0:30 h–1 d (5 timepoints)	[84]
**Bone marrow**	*S. aureus* after 3 h *postmortem* ↓ to 0, ↑ after 5 h until max. at 7 d and ↓ after 14 d until 0 at 30 d Until 24 h, Propionibacteriaceae, *Staphylococcus*, *Propionibacterium*, *Enterococcus*, *Pseudomonas* were detected; at 7 d, *Clostridium* dominated with *Peptostreptococcaceae* spp. and *Pseudomonas*	Mice (*n* = 63); 1 h–30 d (7 timepoints)	[62]
**Mesenteric lymph node**	↑ *Clostridium* sp., *Enterobacter* sp., *Bifidobacterium* sp., *Bacteroides* sp.	Human (*n* = 33); 1–7 d (3 timepoints)	[85]
	Culture-positive until 30 min, ↓ at 1 h, ↑ to max. at 48 h and 72 h. At 5 min, lactobacilli have translocated, ↑ until 30 min, ↓ at 1 h, and then ↑ At 12 h culture + for lactobacilli (high levels), *E. coli*, enterococci, *Bacteroides/Prevotella* spp., clostridia	Mice; 0–72 h (10 timepoints)	[83]
**Uterus**	↑ Alpha diversity; Dominated by Clostridiales and Lactobacillales ↓ Relative abundance of MLE1-12 (*Candidatus Melainabacteria*)	Human (*n* = 40); 24–432 h	[19]
**Prostate**	↑ Alpha diversity Dominated by Clostridiales and Lactobacillales ↓ Relative abundance of MLE1-12 (*Candidatus Melainabacteria*)	Human (*n* = 40); 24–432 h	[19]

^a^ Bacteria phyla are designated according to the List of Prokaryotic names with Standing in Nomenclature (LPSN) and National Center for Biotechnology Information (NCBI): Pseudomonadota (former Proteobacteria), Bacillota (former Firmicutes), Actinomycetota (former Actinobacteria), Bacteroidota (former Bacteroidetes) and Deinococcota (former Thermi). Arrows indicate the increase (↑) or decrease (↓) in bacterial counts throughout time. Abbreviations: ASVs, absolute sequence variants; max., maximum; w, weeks.

##### Gastrointestinal Tract

Dominant normal gut bacteria from the phyla Bacillota and Bacteroidota start changing in abundance and diversity: closely related bacterial species from the Bacteroidales order (e.g., *Bacteroides* spp.) significantly decline over time, whereas Clostridiales (*Clostridioides* spp., *Anaerosphaera* spp.) and Lactobacillales (*Enterococcus* spp.) within phylum Bacillota increase [9,70]. Liu et al. [86] proposed significant biomarkers for gut *Lactobacillus*, *Dubosiella*, *Enterococcus*, and *Lachnospiraceae*. At the later decomposition stages, fecal/rectal samples are dominated by Bacillota and Actinomycetota despite starting/new communities including a high abundance of Bacteroidota (more than skin/mouth). Reports of an increase in Actinomycetota in the drier phases of decomposition are also available [79]. Less dominating Gammaproteobacteria bacteria in live, belonging to the Pseudomonadota phylum (e.g., *Acinetobacter* spp., *Ignatzschineria* spp.), also become more abundant, but the increase seems less consistent between individuals [75,79]. In this case, some bacterial genera (*Ignatzschineria* spp., *Wohlfahrtiimonas* spp.) have been previously identified in flies or fly larvae visiting the bodies, highlighting the contribution of insects on carrion in the evolution of microbial communities during decomposition. Other environmental bacteria such as *Acinetobacter* spp. have been commonly found in soil and dry cadavers [79,87]. Although presenting some inter-individual variability, DeBruyn et al. suggested specific bacterial genera as potential PMI biomarkers linked to increase (*Clostridia* and *Anaerosphaera*) or decrease (*Bacteroides* and *Parabacteroides*) in abundancy during *postmortem* time [70]. The finding of specific bacteria such as *Clostridium* in the end stages of decomposition is not surprising, since it produces amylases and lipases, converting carbohydrates and lipids into organic acids plus alcohols and facilitating fat hydrolysis. The breakdown of proteins in a cadaver is facilitated by various proteolytic bacteria, including *Pseudomonas*, *Bacillus*, and gut sulfate-reducing bacteria. However, during the later stages of decomposition, such as end-bloat, certain bacteria such as *Pseudomonas*, which require oxygen to survive, are replaced by other anaerobic bacteria (e.g., *Clostridium*). This shift occurs due to the reduced redox potential resulting from the absence of oxygenated blood, creating a favorable environment for the growth of anaerobic bacteria [78]. In particular, Guo et al. reported an increase in Pseudomonadota (mostly Gammaproteobacteria) and a gradual decrease in Bacillota and Bacteroidota in the mouse rectum [11].

##### Regarding Skin and Mouth

Pseudomonadota accounts for the greatest biomass before bloat (first 48 h), but Bacillota (skin and mouth) and Actinomycetota (skin) increase in the later stages of decomposition [3,79].

Skin. In forensic investigations, the skin is the most analyzed sample in microbiome studies, including in the sub-nail. The cutaneous microbiome in the palm of a cadaver’s hand remains stable up to 60 h after death and is unique, as only 13% of the bacteria is shared among individuals [12,36]. This opens the possibility of establishing a connection between individual identification and PMI estimation. In addition, the commensal bacteria found in the skin are highly resistant to environmental stress, such as humidity and ultraviolet radiation [12,36]. Indeed, Huang et al. [88] found that skin was the best microbiome at yielding predictions of age in adults in agreement with forensic studies showing that the skin microbiome predicts PMI better than microbiomes from other body sites.

Mouth. In contrast to other sites (e.g., rectum), bacterial populations usually found in life in the buccal cavity seem considerably different immediately after death [11]. The authors described a gradual decrease in Bacillota and Bacteroidota in parallel with an increase in Pseudomonadota (mostly Gammaproteobacteria). After the swollen state, intestinal bacteria, such as *Tenericutes*, can be found in the mouth, which may reflect the migration of bacteria populations from the large intestine [12,36,72]. In the first study using human cadavers as models, Hyde et al. [46] reported differences between two cadavers in the pre-bloat and post-bloat oral communities, but in both cases, *Clostridium* spp. were present in the post-bloat stage.

##### Brain, Heart, Liver, Spleen and Kidney

Limited research exists regarding *postmortem* microbial succession in internal organs, which are presumed to be sterile [53]. However, studying the microorganisms present in internal organs associated with corpse decomposition is of utmost importance, because the presence/absence and the abundance of certain bacteria in these organs can potentially serve as bioindicators of early PMI. In forensic practice, estimating PMI accurately, particularly during the early stages, holds significant value, as it enhances case detection efficiency. Historically, there has been a belief that microbial growth in certain organs, such as the heart, spleen, liver, and brain, occurs only after 24 h *postmortem* [89]. Tuomisto et al. [85] showed that the liver was one of the most sterile samples up to 5 days *postmortem*, after which single isolates of *Clostridium* sp., *Streptococcus* sp., *Enterobacter* sp., *Enterococcus* sp., *Escherichia* sp., and *Staphylococcus* sp. were detected in human models. Can et al. [53] demonstrated the earliest detection of microorganisms in the liver from a human cadaver with a PMI of 20 h and in all sampled organ tissues (heart, blood, liver, spleen, brain) from a human cadaver with a PMI of 58 h. Dell’Annunziata et al. [90] analyzed the internal organs of 10 murine cadavers and showed microbial invasion at 3- and 10-days *postmortem* for the liver-spleen and heart-brain, respectively. However, a recent study revealed in mouse models that these internal organs, including the brain, heart, liver, and kidney, can harbor bacteria as early as 0.5 h after death, up to the 24 h *postmortem* evaluated [84]. During this early period, they present a relatively low species richness and abundance of bacteria, the dominant microbial species differ among organs, but they tend to become similar over time. As an example, in brain samples, the abundance of *Acinetobacter* increased significantly around the 8 h mark.

Different studies suggest that microorganisms multiply in blood, liver, spleen, heart, and brain, in a time-dependent manner, and their relative abundances are unique to each organ and PMI, meaning that when samples are analyzed, they tend to group based on the cadaver or PMI rather than the specific organ tissue [53,54,84]. As with other external organs and body sites, the *postmortem* microbial communities within internal organs may experience unique shifts and dynamics, potentially influenced by factors, such as environment and organ-specific conditions.

##### Other Cadaveric Samples

At later stages of decomposition, microbial successions of bones or soil should be the main choices [12]. Emmons et al. [91] demonstrated that the *postmortem* bone microbiome is distinct from the human gut and soil, but with similarities to each depending on the depth of the bone in the soil. The unique conditions surrounding the burial site shape the microbial community that develops within the bones, and *Pseudomonas* and phosphate solubilization seem to play a key role in skeletal degradation. Human and soil-associated bacteria unite to create a unique bone microbial profile after death: bacterial communities at the surface are more like soil and those in buried bones more like the gut (more anaerobic), with conditions such as the depth of human remains influencing the composition of the *postmortem* microbiome. Soil microbiome greatly affects the human *postmortem* microbiome, especially in the late stages of decomposition.

#### 3.2.3. Factors Affecting Decomposition

The diversity and inter-individual variability observed in the *antemortem* human microbiome, which are highly influenced by diet, age, sex, ethnicity, country of origin, comorbidities, and antibiotic use, among other factors, potentially affect the thanatomicrobiome composition and subsequently the microbial succession occurring after death. Several different abiotic and biotic factors, either present *antemortem* or *postmortem*, contribute and directly influence the decomposition process: the abiotic factors include conditions such as time, temperature, humidity, pH, and *antemortem* living habitats (e.g., diet and antibiotics); and the biotic factors include insects, scavengers and *antemortem* infections [75].

Within abiotic factors, time is a crucial one since it greatly affects the abundance and diversity of bacteria over time, playing an important role in estimating the time since death. Also, temperature and water activity (humidity) strongly accelerate the decomposition process by affecting the thanatomicrobiome composition, both qualitatively and quantitatively, and bacterial tissue colonization. The temperature increase has been linked to changes in detritus availability and necrobiome dynamics, with cadaver decomposition evolving faster in hot climates [12,75]. In addition, bacteria can present variable resistance to humidity or other environmental conditions, since different species require variable optimum temperature/humidity for growth (for example skin bacteria are highly resistant to humid contexts), highlighting the role of knowing epinecrotic communities as well. Major changes in pH also occur after death, mainly associated with a pH decrease in blood and gastrointestinal tract, enabling acidophilic bacteria to thrive (e.g., *Cutibacterium acnes* in the skin). However, there are some reports of pH increase in specific body sites [75] being difficult to assess and control all changes induced by the innumerous by-products generated after death. *Antemortem* intake of drugs, namely antibiotics, is one of the major factors influencing the *postmortem* microbiome. Thus, it is crucial to know the medical and epidemiological history of the person in question. It is well known that the prolonged use of antibiotics, for example, disturbs a healthy microbiome, meaning that the thanatomicrobiome will also be affected. However, an increasing number of different scenarios, such as drug overdose cases, for which the *postmortem Clostridium* effect has been described, are being explored [75,92]. Finally, daily diet and lifestyle habits also directly influence the composition and diversity of the gut microbiome, hardening the generation of robust microbiome databases to be used and applied in forensic investigations [18,93].

The decomposition of human carrion is primarily achieved by necrophagous invertebrates (mostly insects) and large scavengers (like other vertebrates such as opossums or vultures), apart from the present microorganisms—the biotic factors [75,94]. The microbial interactions on human remains themselves can dictate which insects are attracted to and colonize them. For example, along the decomposition process, bacteria produce large amounts of gases through fermentation and those volatile chemicals (called apeneumones, e.g., H_2_S, CO_2_, NH_3_) attract many of the invertebrates and vertebrates that help decompose the remains. A series of scavenging activities succeed with the successive attraction of different predators and parasites to survive on the conditioned human remains, such as dry skin, bones, and hair. The physical condition of the dead individual is also key in the decomposition process. A body with a higher amount of fat maintains the inside temperature for longer resisting more to the degradation process. However, at the same time, it provides more nutrients such as nitrogen for bacterial growth, meaning that decomposition starts quickly, but bloating takes longer, a slower mass loss occurs and skeletonization is prolonged. Overall, because it is not linear but multifactorial, smaller carcasses decay significantly faster than large ones and this must be taken into account when testing decomposition-related models and methods to assess PMI [95].

The existing *antemortem* microbial abundance also plays a key role in decomposition. An elderly body has approximately 40 trillion microbial cells and, for this reason, has a much faster decay rate than a fetus or a newborn that eventually dies [19]. The same is true in deaths from infection where the number of microorganisms is obviously higher. When there are nutritional disorders, such as anemia, or death by poisoning, during life, degradation is slower since the environment is not favorable to microbial growth [12,50].

## 4. Microbiome-Based Analysis for Forensic *Antemortem* and/or *Postmortem* Applications

**Figure 1:**
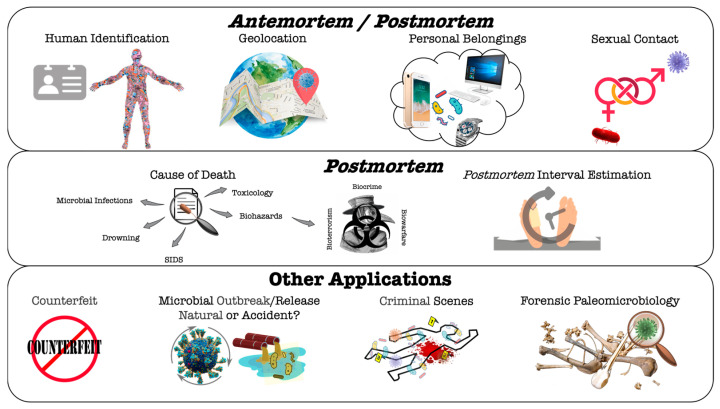
Graphic representation illustrating possible microbial forensic applications to answer criminal/legal cases. *Antemortem* and *postmortem* applications are addressed in more detail in the text. The acronym SIDS stands for Sudden Infant Death Syndrome.

Due to the widespread presence of microorganisms in the environment and intrinsic to the cadaver, bacteriology, and mycology have been applied as a tool for a wide range of forensic techniques [20,35,46,50]. In fact, since the human microbiome project launched in 2007 (https://hmpdacc.org/; last accessed on 30 June 2023), our knowledge about the thousands of microbial species colonizing us has not stopped growing. From a forensic point of view, a particular or a bunch of microorganisms can provide clues as trace evidence in different scenarios (Who? What? When?), from personal identification to cause of death or PMI calculations [96]. The analysis of microorganisms as biological threats (or biothreats) or biohazards, considered as biorisks, referring to the accidental or deliberate release of a pathogen or toxin into a susceptible population (bioterrorism, biocrime, or biowarfare), is not the subject of this review and will not be discussed here [97]. The sections below describe the main applications of microbiome analysis in *antemortem* and/or *postmortem* forensic studies (Figure 1).

### 4.1. Microorganisms or Microbiome Analysis in Ante/Postmortem Forensic Studies

#### 4.1.1. Human Identification

Given the similar or even greater number of bacterial cells compared to human cells in particular body sites, it is conceivable that as many bacterial cells and their genes are deposited in touched items in comparison to human markers. The characterization of the personal microbiome and the microbial transfers noticed between people and objects can be used to identify a suspect when their bacterial community is left at the scene of a crime or directly on the victim. This is achieved through the characterization of microorganisms in the sample, enabling the identification and correlation of the microorganisms present with the sourced tissue, and taking into account the tissue’s unique structure and composition, according to the area concerned, on the intervener and on the geolocation [18,20,98]. Existing transfers can be classified as direct transfers between humans and objects (for example, through a handshake or between the site and the body), or indirect transfers that occur between humans, using an object as a bridge. However, the applicability of this method in forensic sciences requires a high preciousness to avoid contaminations [20]. Many studies on the skin microbiome suggest that the palm microbiome has significant potential as a long-lasting “fingerprint” for human identification, especially when objects remain untouched for months. However, dominant skin species, such as *Staphylococcus epidermidis*, may be less suitable as biomarkers compared to minor species, as the latter can be linked to specific individuals [99,100,101]. An idea gaining strength is the combination of human microbiome analysis with traditional human DNA testing (e.g., short-tandem-repeat [STR] analyses) to provide complementary data for stronger associations and exclusion of individuals falsely associated with biological evidence [102]. For example, by using a new tool for saliva identification with three oral bacterial markers, Jung et al. verified the existence of specific oral bacteria in 91.4% of samples with high sensitivity (with very low DNA levels and with residual effects after tooth brushing) and specificity (by comparison with fecal samples) [103].

#### 4.1.2. Geolocation

Previous studies carried out on the human microbiome have revealed the variations that exist in the microbial ecology of different populations on our planet. These differences may be due to distinct factors, such as the level of industrialization in each geographic region and/or the lifestyle of each population, which has increased the forensic interest in finding microbial signatures that characterize each geographical area. Indeed, microbial populations are highly dependent on their geographical location, which is directly affected by variations in altitude, latitude, climatic conditions, and soil composition [20,104]. In this context, the Earth Microbiome Project was created in 2010 (https://earthmicrobiome.org/; last accessed on 30 June 2023) to sample the whole planet’s microbial communities and, thus, to assess biogeographic variations of microbial communities. Each city shows unique microbial profiles contributing with high accuracy to the geographical identification of the place of death or surrounding areas [105]. Available studies exploring the use of microbial profiles for geolocation showed, among other promising data, clear differences in the most common species in people from different cities in different countries. They also showed that scalp hair samples seem to more robustly predict geolocation than pubic hair samples (a result of great forensic relevance), and that gut microbiota differs significantly between European, North American, Japanese, Korean, and Colombian populations [106,107,108]. An important constraint of using the microbiome to determine geographic origin is that microbial indicators associated with location can vary by interacting with new environments or by sudden changes in a person’s lifestyle. This indicates that longitudinal studies should evaluate different variables.

#### 4.1.3. Personal Belongings

Humans have a unique skin microbiome that is generally stable over time and transfers to objects they interact with, generating a microbial signature on personal objects and surfaces that is stable for forensic scenarios. Different studies, both *antemortem* and *postmortem*, in corpses were able to link microbiomes of hands/shoes with those present on objects/surfaces, including different geographical areas [98,109,110]. Interestingly, particular bacteria from the donor’s hands could be correlated with lifestyle, estimation of gender, and ethnicity (e.g., the absence of *Alloiococcus* indicated female gender, Asian ethnicity, and use of hand sanitizer), corresponding to personal features of a potential large forensic relevance. The finding of stable skin microbiomes from corpses and personal items during transport and storage in the morgue is promising whereas the precision of results varied between the different objects (e.g., mobile phones, glasses, etc.) analyzed [106]. Naturally, there are some difficulties in using such data since the microbiome of hands and footwear changes over the day and the same occurs for the microbiome of floors and other surfaces that may alter depending on how many people walk/touch it. In *postmortem* studies, in particular, it remains unknown how long the time period is when the human skin microbiome is no longer viable as a personalized signature [64].

#### 4.1.4. Sexual Contact

For years molecular/serological identification has supported investigations of sexual assault by identifying unusual pathogens (absent from the human microbiome) in the victim and the suspect (e.g., *Neisseria gonorrhoeae*, *Chlamydia trachomatis*) [111]. More recently, and owing to the improvement of sequencing technologies, phylogenetic analysis is able to identify with higher precision (at the strain level) the pathogens potentially transferred in sexual assault cases, including with several possible victims [112]. Now we are entering a different era where the entire microbiome of both the suspect and victim(s) can be explored. Given the uniqueness of the microbiome from person to person and that microbiomes of couples with sexual contacts tend to be more similar to each other than to those of unrelated people, the occurrence of some transfer during sexual contact may allow the human microbiome (genital and/or pubic hair) to be used in investigations of sexual assault when there is no other evidence. The most robust studies showed the stability of the pubic microbiome for 6 months, including at variable storage times and temperatures, which seems not to be influenced by an increased frequency of sexual activity, but by particular gender differences (Bacillales or *Corynebacterium* are more abundant in men, while Bifidobacteriales and/or Lactobacillales can be more abundant in women) [113,114,115]. Moreover, in cases of sexual assault, the evaluation of a woman’s microbiome predicted with high accuracy the expected proportion of the aggressor when a single suspect or a small group of suspects were investigated.

### 4.2. Microorganisms or Microbiome Analysis in Postmortem Forensic Studies

#### 4.2.1. Cause of Death

Different studies established the microbial communities in association with the cause of death and found that the beta dispersion differed significantly between anatomical body sites and modes of death, namely dysbiosis, drowning, or sudden infant death syndrome [12]. The presence of specific microorganisms can act as evidence or bioindicator for the cause of death, which may be useful in confirming the diagnosis of an *antemortem* infection or a previously undiagnosed infectious disease, or in identifying microbial markers for particular types of death [8,83]. It has been proven that the different etiologies of death, including natural, accidental, suicide, and homicide, also significantly influence the distribution of microbial communities in the different regions of the cadaver [19]. Though so far clinical microbiology hasn’t started using microbiome techniques to identify infectious causes of death, sequencing tools to characterize human microbiomes could be of use in the future in the medico-legal field.

##### Hospital/Community-Acquired Infections and Other Biorisks

A manifold of reasons can contribute to microbiota disequilibrium (dysbiosis) and this may not always be easy to distinguish from the changes that occur in the human microbiome after death. Generally, the detection of a single microbial species in body fluids, especially those leading as causal agents of hospital-acquired or community-acquired human infections (such as *Streptococcus pyogenes*, *Streptococcus pneumoniae*, *Staphylococcus aureus* or *Candida albicans*), indicates an *antemortem* infection. By contrast, a mixed profile points to a *postmortem* invasion, as a consequence of a contamination from the skin or intestine [22]. However, a true pathogen can be isolated more frequently in a mixture with other contaminants. The presence of *Propionibacterium acnes*, *Corynebacterium* sp., or *Bacillus* sp. (except *B. anthracis*), is much less often associated with an infection [50].

Chronic diseases, for example, can have a significant impact on the human microbiome, which may occur as a result of changes in the host’s physiology or lifestyle or as a consequence of medical treatments, such as antibiotics. In forensic sciences, the understanding of the relationship between chronic diseases and microbial dysbiosis can be important in reconstructing the medical history of a deceased individual and in determining the cause of death. In the early stages of liver disease, there is an increased intestinal permeability, which occurs independently of changes in the microbiome or endotoxins. Cholestatic liver injury is characterized by a translocation of bacteria from the families *Enterobacterales*, *Enterococcaceae*, and *Bacillaceae* primarily to the mesenteric lymph nodes [116]. In cases of death from cardiac arrest, there are sex differences in the populations of microorganisms. *Clostridium* spp. and *Streptococcus* spp. are highly abundant in men and Clostridiales and *Pseudomonas* spp. in women. This demonstrates that the hearts of males and females form distinct bacterial niches after death [117]. In another study, it was shown that a decreased phylogenetic diversity represents a highly significant predictor of heart disease, with a dominant presence of *Streptococcus*, *Prevotella*, *Fusobacterium*, and *Rothia* [72].

Hospital-acquired infections (HAIs: infections that first appear 48 h or more after hospitalization or within 30 days after having received health care) or nosocomial infections are a common cause of death even in industrialized countries with advanced healthcare systems. The World Health Organization (WHO) estimates that 1.4 million people suffer from HAIs worldwide and that particular pathogens are more often involved in these scenarios (e.g., *S. aureus*, *S. pneumoniae*, *E. coli*, *K. pneumoniae*, *C. albicans*), some of which belong to the human healthy microbiome. As most of them are opportunistic pathogens, taking advantage of particular situations, namely patients with dysbiosis from chronic diseases, elderly people, invasive surgeries, immunosuppression, etc., strict preventive and hygienization measures followed by healthcare institutions are crucial to avoid outbreak and human transmission events through contact with contaminated medical equipment, surfaces, or healthcare workers. In this context, microbial forensics is important for clinical diagnostics and public health protection, for example in the event of an infectious disease outbreak scenario in which the accurate identification (ideally until the strain level) of the agent is highly relevant, namely for hospital tort litigation lawsuits by patients against hospitals arising from a HAI [35]. Naturally or community-acquired infections (e.g., pneumonia) are infections contracted outside of a healthcare setting, originating naturally from an individual event of dysbiosis/immunosuppression or spread through contact with infected people, contaminated objects, or the environment. They need to be distinguished from those that result from malicious transmission and, in all cases, infections can be traced back to the patient’s activities, environment, and contact with others together with genotyping/sequencing data from human and environmental/clinical samples to determine the source of the infection. The final goal is always to determine the source of the infection, the method of spread, and the parties responsible, in order to prevent future infections and hold those responsible accountable. As we are facing an unprecedented revolution in sequencing and other analytic methods, we never know what microbiology still reserves under this context; for example, by combining laser microdissection and 16S rRNA sequencing, osteomyelitis caused by *Pseudomonas aeruginosa* was identified as the cause of death of a child from the 18th century [118].

##### Drowning

Drowning is a usual cause of death for victims recovered from watery environments and determining this type of death is, whenever possible, based on pathological findings. Diatoms are aquatic algae from the phytoplankton and, as long as their density is high enough, their analysis can be useful to estimate the type and amount of diatom-rich water aspirated into the lung before death. This approach is limited if diatoms are too large and they cannot reach the bloodstream and organs if they are present in low concentrations, or simply because they can also be found in non-drowned bodies (false positives) [119]. For these reasons, smaller aquatic microorganisms (bacteria, cyanobacteria, and bacterioplankton) present in large amounts have been increasingly explored as markers of death by drowning [106]. The established knowledge of the usual aquatic habitats of particular microorganisms, such as *Aeromonas* in freshwater, *Vibrio* and *Photobacterium* in saltwater, and all three genera in brackish water, facilitates their future use as markers to support the diagnosis of drowning deaths [120]. Kakizaki et al. [121] demonstrated, by culturomic experiments, that the detection of bacterioplankton in a blood sample may support the assumption of death by drowning since commensal bacteria do not readily invade the bloodstream after drowning. As so, the location and circumstances of the drowning can be established after identification of the bacteria present in the water where the drowning occurred, which can be compared to bacteria found in a victim’s lungs to determine if the victim was indeed exposed to that specific water source. These findings, together with other medico-legal investigations that include strontium [122], other chemical markers, diatoms, and histopathology as well as the victim’s medical history and the circumstances of the incident, must also be taken into consideration in determining the cause of death.

##### Sudden Infant Death Syndrome (SIDS)

SIDS is defined as the sudden and unexpected death of an infant <1 year during sleep that remains unexplained after a thorough investigation that includes the examination of the site and circumstances of death, a complete autopsy, the revision of the clinical history of the victim and their relatives, and an interview with the parents. The complete autopsy should include ancillary analyses: histopathology, microbiology, toxicology, and biochemistry among others. but there is growing evidence that microbiota dysbiosis may play a role in SIDS [123]. Available studies indicate greater colonization of SIDS cases with different species of *Clostridium* and Bacteroides, babies sleeping in a prone position seem to be more heavily colonized with *S. aureus* and a significant association is shown between SIDS cases and infections by bacterial and viral pathogens [124,125]. An alternative hypothesis relies on the development of the infant gut microbiome after birth in interaction with the brainstem serotonergic system [126].

##### Toxicological Effects Imposed by Microbial Metabolism

*Postmortem* microbial activity may interfere with autopsy toxicological results. It is known that during decomposition some microorganisms are responsible for the degradation of drugs (e.g., antidepressants, benzodiazepines, cannabinoids, cocaine) or other xenobiotics (e.g., cyanide) and for the neoformation of other metabolites (e.g., alcohol, methamphetamines and amphetamines, opioids) [35]. This is of particular concern if the body has been exposed to higher temperatures favoring microbial growth since it increases the corpse’s degradation prior to autopsy. Some remarkable examples include the bioconversion of nitrobenzodiazepines (e.g., clonazepam, diazepam) into their 7-amino-metabolites by several species (e.g., *Bacillus cereus*, *Staphylococcus epidermidis*, *Clostridium perfringens* and *Bacteroides fragilis*); the *N*-demethylation of methamphetamine into amphetamine by intestinal bacteria (e.g., enterobacteria, enterococci, *Lactobacillus*, *Clostridium*); or the production of ethanol or other byproducts, from sugars, amino acids, fatty acids, among others, by a variety of bacteria (*Corynebacterium* spp., *Escherichia coli*, *Enterococcus faecalis*, *Klebsiella* spp.), yeasts (e.g., *Candida* spp, *Saccharomyces cerevisiae*) and fungi (e.g., *Aspergillus* spp.). Ethanol is a well-explored example and, even though forensic toxicologists have established ethanol values in blood for different situations, determining whether blood ethanol concentrations originate from *antemortem* ingestion or from *postmortem* production can prove to be very difficult even using preservative agents. This is particularly relevant in advanced cases of decomposition, which may be favored by warm and moist environments [127]. Other circumstances may be taken into account when evaluating a possible ethanol intoxication scenario. For example, auto-brewery syndrome, which is a rare and an underdiagnosed condition, implies a gut microbiota dysbiosis in which illegal levels of ethanol are detected in the blood even without ethanol consumption [128]. As such, the deep and increasing knowledge of the human *antemortem* and *postmortem* microbiome can provide a full snapshot of the microorganisms present in different organs and body sites, and so a better understanding of the changes in drug metabolism occurring after death (either by degradation, production of bioconversion) and how they may impact autopsy and the interpretation of toxicology *postmortem* results.

#### 4.2.2. Estimation of *Postmortem* Interval

The estimation of the PMI, i.e., the time elapsed since death, is probably the most studied application of the *postmortem* microbiome and a fundamental part of a criminal investigation [3,80]. There are pieces of evidence that *postmortem* microbiomes do not significantly change within 24–48 h of death and can reflect *antemortem* health status [72]. In fact, *antemortem* conditions, such as drug/ethanol or another stressful lifestyle (e.g., dumping) linked to homicide, for example, greatly influence *postmortem* microbiome composition [129]. Despite the existence of a great diversity of methods with the potential to predict, in a certain way, the PMI, their accuracy remains limited and none of them can be recognized as an accurate and universal tool. The selection of one to the detriment of another is based on each case, on the available data, and the particular circumstances of each case. Thus, in order to get a more rigorous prediction, several methods have been employed in parallel, thus reducing the margin of error of each method individually.

##### Evolution of Methods for PMI Estimation

**Figure 2:**
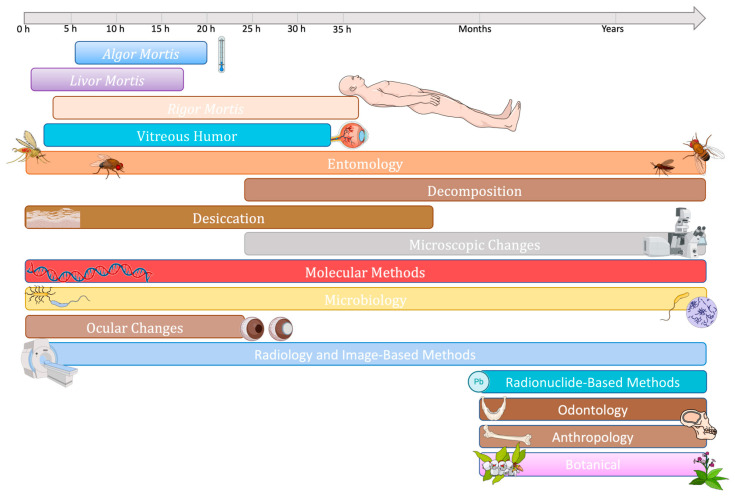
Graphic representation illustrating the main methods to estimate PMI and the time frame within which can be employed (adapted from [133]). Algor mortis, the cooling of the body after death, is probably the most widely used method for estimating PMI and is based on the decrease in rectal temperature [68]. Livor mortis corresponds to the change in skin color due to the deposition of stagnant blood in the lower parts of the body after death and is assessed by the color and fixation of lividity through the bleaching test [68,134]. Rigor mortis, also called *postmortem* rigidity, represents the third stage of death characterized by the stiffening of muscles due to the binding of actin and myosin filaments (starts around 2–3 h after death) [68]. The vitreous humor, a clear and colorless fluid that fills the space between the lens and the retina of the eye, offers an alternative biological matrix for estimating the PMI through a valuable biochemical method [12,135]. Entomology, the study of insects and their interactions with other organisms, humans, and the environment, has been used for forensic purposes since the 13th century, providing insights into PMI through the analysis of insect colonization and life cycle evolution on decomposing cadavers [136]. The process of decomposition after death involves the degradation of soft tissues through autolysis (cellular breakdown) and putrefaction (bacterial consumption), with various factors influencing its progression; there is a somewhat predictable sequence of stages, including fresh, bloat, decay, and dry [134]. Desiccation, the process of drying of the skin and mucous membranes after death, leads to changes in color and texture, with notable effects observed in the eyes as well as in the skin where the lips and genitalia are particularly affected [134]. Microscopic changes, including alterations in tissue histopathology, immunohistochemical, and protein densities, can provide valuable information for estimating the PMI based on the decrease or increase in specific proteins and compounds [134]. Molecular methods, including RNA and DNA analysis, offer valuable tools for PMI estimations by assessing nucleic acid integrity, measuring degradation rates, and quantitatively amplifying target genes, although DNA degradation poses limitations for longer PMIs [134]. Microbiology, the main focus of this review is discussed in detail in the next section. Ocular *postmortem* changes include variations in corneal opacity, pupil diameter, blood vessel striation, retinal color, and intraocular pressure [134]. Forensic radiology is an emerging area of forensic sciences that deals with the utilization of imaging techniques such as radiography, computed tomography, and magnetic resonance imaging for the examination and analysis of deceased individuals [134]. Radionuclide-based methods, commonly employed in forensic anthropology for skeletonized remains, utilize radionuclides such as 210Pb and 210Po, 14C radiocarbon, as well as measurements of citrate and nitrogen content [134,137,138]. Forensic odontology uses dental evidence to provide expert analysis and answers to forensic inquiries, serving both the field of justice and anthropology [139]. Forensic anthropology analyzes bone evidence, whether fragmented or intact, to respond to the law in several aspects, including identification, gender or age estimation, and PMI calculation [137]. Through botanical classification and traditional knowledge of plant species, the relationships between their development, specific habits, and geographical origins can be established, adding valuable information about the environment surrounding a corpse and possibly in the PMI estimation [140].

Several methods have been implemented over the years to estimate PMI (Figure 2) [130,131]. 

The PMI estimation is primarily based on the visual inspection of the cadaver (e.g., *algor*, *livor*, and *rigor mortis*) in early *postmortem* periods, which are the standard tools in routine practices, and on alternative methods such as histopathological and biochemical methods that remain relatively imprecise. In later *postmortem* periods, PMI calculations with such classical methods are harder to apply and other methods as forensic entomology and molecular tests may be more relevant [12,131]. Forensic entomology has provided a reliable evidence-based alternative for PMI calculation based on invertebrates’ biomarkers found in the cadaver or the surrounding environment [10,11,12,50,64,132]. Studies on changes in the bacterial communities of internal organs, aiming to establish a relationship between bacterial interactions and the time elapsed after death (the so-called microbial clock), have been increasingly employed in the PMI calculation. This relies on the fact that bacterial communities experience a dynamic alteration over time after death, showing, therefore, potential as biomarkers for PMI calculation [19,36,62,70].

##### Microbiome, Microbial Communities or Microbial Succession

Bacterial predictors are generally related to the different events of cadaveric decomposition. Particularly, in the calculation of the PMI the microbial clock is more accurate in the first 48 h after death, as the cadaver is still in a fresh stage with limited contamination by soil microorganisms. Even though there are some descriptions of PMI estimates errors varying from 2 to 7 days [10,20,64], the accuracy and precision of PMI estimates is unknown, as error can arise from sources of variation, such as measurement error and environmental factors. Microorganisms actively engage in decomposition owing to their ability to use available nutritional sources and their inherent resistance features. Furthermore, in contrast to human cells, bacteria possess a circular shape and robust cell walls, affording them protection against degradation [8,12,20,36,64]. It is in the decay period, in particular, that a fundamental role of bacterial action in the decomposition of the human body is verified [65].

Table 1 comprises the current knowledge about the fluctuating microbial communities along cadaver decomposition with the different studies using different strategies/technologies essentially varying in (i) the organism used (human, mouse, swine) and its individual characteristics (e.g., sex, age, weight); (ii) the DNA extraction method and sequencing technology; (iii) the sampling method; (iv) the time points tested; (v) the body sites tested even within the same organ (e.g., skin); and (vi) the number of samples and replicas. Although the influence of these variables on changes in microbial communities is not well established, available studies suggest that succession of bacterial communities may be used to estimate PMI, since they could associate common bacterial groups (predominately bacteria from Gammaproteobacteria, Lactobacillaceae, and Clostridiaceae) with decomposition corpses [53,67]. Therefore, and as aforementioned within Section 3.2.2, particular bacterial phyla, families, and/or genera are key *postmortem* taxa involved in the body decomposition process. Particular bacterial families or genera have even been proposed as potential PMI indicators, such as those belonging to Pseudomonadota and Bacillota. Javan et al. [54] proposed some species from the Bacillota phylum (*Clostridium* sp., *Bacillus* sp., *Peptoniphilus* sp., *Blautia* sp. or *Lactobacillus* sp. strains) as potential biomarkers in estimating time of death. Adding to the challenges of definitively identifying bacterial biomarkers is the description of contradictory reports across different studies (e.g., an increase versus decrease in bacterial richness; Table 1). These inconsistencies can be attributed to variations in study models, the inclusion of different body parts, the intricate microbial and metabolic networks involved, and the multitude of factors influencing human body decomposition.

In one of the very first studies addressing PMI using gene markers by high-throughput sequencing, Pechal et al. [3] used swine remains over 5 days to describe that the ability to differentiate bacterial communities throughout physiological time depends on the level of taxonomic resolution. While the best results were observed using a model built on the four phyla (Bacillota, Bacteroidota, Pseudomonadota, Actinomycetota), specific bacterial families, including *Campylobacteriaceae*, *Enterococcaceae*, *Moraxellaceae*, *Prevotellaceae*, and *Pasteurellaceae*, retrieved even better results. By using a similar approach by Pechal et al. [3] with building regression models informed by the Random Forest classifier but in a mouse model, Metcalf et al. [80] showed that PMI can be estimated within 3 over 48 days (with the highest accuracy before 34 days) and that combining skin and soil microbial communities (16S rRNA and 18S rRNA datasets, respectively) provided the most accurate PMI prediction models, at least compared to the abdominal cavity where during the rupture stage microbial changes are more variable. For bacteria, taxa in the order *Rhizobiales* (Pseudomonadota) were among the most important predictive taxa at each sampling site. In a follow-up by the same authors [4], they found the greatest PMI accuracy (2–3 days over the first 25 days of decomposition) from microbial samples of the caecum, soil, and skin over the first 2 weeks of decomposition, and that the soil type did not affect the accuracy of the “microbial clock”. Another remarkable exciting result was achieved by Metcalf et al. [4] describing accurate time estimates since placement across different seasons, meaning an advantage in using microbial communities that can circumvent some of the factors affecting body decomposition and entomology tools [7]. Even after the corpse has completely decomposed, the microbial signatures belonging to the body remain in the soil for months or even years, allowing clandestine graves to be found [7].

Since these first descriptions, an increasing number of studies describe potential microbial signatures or biomarkers of human decomposition suitable for *postmortem* calculations (Table 1; [106]). Most studies used 16S rRNA gene sequencing to detect a wide range of bacteria with the limitation of generally reaching only phyla or family taxonomic levels. Independently of the methodological approach, different studies showed *Clostridium* as a strong positive predictor of PMI [70,78,141]. *Clostridium* species are known to play a crucial role in facilitating decomposition by breaking down lipids and complex carbohydrates associated with human tissue. *Clostridial* lipases are believed to significantly aid in fat hydrolysis, particularly in hot and humid environments characterized by low oxygen levels and limited redox conditions, while their hydrolytic enzymes convert carbohydrates into organic acids and alcohols, further contributing to the decomposition process (the *postmortem Clostridium* effect). When the body is recovered from water, the time of death is often defined by the *postmortem* submersion interval (PMSI), which is the time elapsed since the body was immersed in water. The marine barnacles *Notobalanus decorus decorus* have been used as promising bioindicators [50], but we do not explore PMSI and microbial communities in this review.

## 5. Methods and Technical Issues

Microbiological analysis can be carried out by two distinct metagenomic techniques: culture-dependent or by genetic approaches. It is important to note that these methods are commonly applied to daily situations, ranging from microbe identification in hospital routine, outbreak resolution, food quality control, and academic research, among others. Depending on the final goal and available resources, one or several methods can be simultaneously employed, from classical microbiology (e.g., culture media, biochemical tests) to high-throughput sequencing methods [12,142].

### 5.1. Culture-Dependent Methods

The culture dependent-method relies on cultivating bacterial species in specialized microbiological media, enabling the isolation of viable microorganisms from samples. This approach provides a means to characterize these microorganisms with a high degree of accuracy and taxonomic resolution [52]. While this method is relevant in forensic analysis due to its ability to precisely identify taxa, it does have limitations, including the prolonged cultivation periods required by some microorganisms [50]. In addition, considering that approximately 99% of environmental bacteria cannot be successfully grown in laboratory conditions and that up to 80% of bacterial species inhabiting the human body are considered “unculturable”, the identification of bacteria using these methods can present a challenge in the forensic analysis [11,46,52].

Na et al. [143] examined *postmortem* body fluid samples from human Korean autopsy cases to identify various bacterial genera and species that are typically part of human normal microbiota together with C-reactive protein testing to identify the presence of *antemortem* inflammation. However, the authors employed a combination of genetic and biochemical tests for bacterial identification, resulting in a lack of a definitive and comprehensive list of results. Tuomisto et al. [85] analyzed 33 human autopsy cases by bacterial culturing and concluded that liver and pericardial fluid were the most sterile samples up to 5 days *postmortem* (the latest being invaded). This research has also shown that the relative amounts of intestinal bacterial DNA (Bifidobacteria, Bacteroides, enterobacteria, and Clostridia) increase with time. Burcham et al. [82] used reinforced clostridial medium and mannitol salt agar to, respectively, count colony growth and analyze *postmortem* dynamics of *Clostridium perfringens* and *Staphylococcus aureus* in a mouse model. Dell’Annunziata et al. [90] analysed, on 10 murine cadavers, microbiological swabs from external anatomical sites and internal organs during 16 and 30 days. The resulting swabs were plated on blood, MacConkey, Chocolate, Sabouraud, and Schaedler agar plates, allowing the authorsto infer that the initially sterile internal organs showed signs of microbial invasion at 3- and 10-days *postmortem* for the liver-spleen and heart-brain, respectively, and the *postmortem* microbiota was mainly dominated by Bacillota and Pseudomonadota.

Despite the limitations associated with culturomics, its accessibility in routine microbiology makes it a valuable tool. Further studies exploring this field can yield important insights into key bacteria or identify useful biomarkers derived from these bacteria with the potential to be incorporated into routine analyses using simple methods.

### 5.2. Culture-Independent Methods

Current methods for the genetic profiling of the thanatomicrobiome greatly aim to elucidate the microbial communities present in a given sample (often called thanatogenomics). Microbial community sequencing is achieved by two methodological approaches: (1) amplicon (marker gene) sequencing, a method that amplifies variable regions of a highly conserved bacterial gene, such as the 16S rRNA gene, enabling to infer taxonomic/genomic relationships based on their phylogeny; and (2) whole genome shotgun sequencing or metagenomics, an approach that sequences all (“meta”) of the DNA present in a given sample [144]. It is important to highlight that analyzing specific gene markers after sequencing (e.g., the 16S rRNA gene) is not metagenomics (a common literature misinterpretation) as this corresponds to gene amplicon sequencing. These methods are based on the extraction of genetic material from samples by using DNA commercial extraction kits that greatly facilitate the process, and represent rapid, precise, and very informative methods for identification of bacterial populations.

Regarding amplicon sequencing, the most common marker genes include the 16S rRNA gene for prokaryotes (bacteria and archaea), the 18S rRNA gene for eukaryotes, and the internal transcribed spacer for microscopic fungi [52]. Most available studies explored in this review addressed microbial taxa by the bacterial genetic marker 16S rRNA gene. This can provide in-depth coverage due to the relatively short sequence with hypervariable regions appropriately sized for the sequencing platforms. In addition, given its conserved regions, it allows for designing universal primers to target the hypervariable regions that denote the variable microbial diversity. Moreover, 16S rRNA sequencing provides consistent and longitudinal results, is cost/time-saving, and is suitable for large numbers of samples simultaneously for estimating the time and location of death [145]. The V4 hypervariable region seems the most sensitive for microbial signatures, while the overlap between the V2 and V4 hypervariable regions shows the highest accuracy for taxonomic determination. Nevertheless, the greatest limitation is that 16S rRNA gene profiling has low discriminatory power for some bacterial groups, enabling the identification of bacteria until the genus level, occasionally until the species level. Nevertheless, this limitation can be bypassed by sequencing the entire 16S gene and the intragenomic variation between 16S gene copies [146]. Metagenomics is, on the other hand, able to provide strain-level characterization by producing sequence reads of strain-specific markers, and to quantify the taxa diversity within the sample analyzed (α-diversity) as well as the diversity among distinct samples (β-diversity) [12,50,52,147]. Different methods and bioinformatic tools, requiring efficient and accurate computational pipelines besides robust and updated reference databases, have been applied to profile the human thanatomicrobiome communities. However, it poses a big current challenge given the massive scale of data generated [12,50,148,149,150]. The future may rely on machine learning methods (boosted algorithms, random forests, neural networks, or new ones that may appear) for modeling *postmortem* microbiomes, overcoming the different analytical challenges, and performing reliable predictions in death investigations [151].

Other approaches involving transcriptomics and proteomics, which provide functional community information and/or a global snapshot of the physiological and biochemical state of a sample, can be highly valuable (e.g., when DNA is absent or degraded as in hair or bone samples) and are increasingly explored [152,153]. Also, flow cytometry (evaluating DNA degradation) and MALDITOF-MS (Matrix-assisted laser desorption/ionization time-of-flight mass spectrometry) or MALDI-IMS (Matrix-assisted laser desorption/ionization imaging mass spectrometry), respectively able to accurately identify bacteria at the species level and profiling the proteins and peptides in tissue sections, have been explored as promising tools for PMI calculation [71,154,155].

## 6. Advantages and Limitations of Microbiome Analysis in Forensic Investigations

It is currently undeniable that microbial markers may complement routine forensic tests. The huge technological advances over the last two decades made it possible to overcome significant challenges such as identifying unknown or hoax microorganisms, either non-cultivable in the laboratory, present in low numbers, in complex matrices or even genetically modified, or in cases where samples are degraded and human cellular components are limited and where the abundance and resistance of microorganisms may be an advantage [12,96]. Current methodological approaches allow faster diagnostics and monitoring independent of culture media, the reduction of time and associated costs, and surveillance of outbreaks and epidemics in real time. An extraordinary example is the fast and real-time vigilance and monitoring during the COVID-19 pandemic. The management of this crisis was only possible due to the current sequencing and bioinformatic technologies we have nowadays available in many countries, enabling to identify and characterize, at the strain (SARS-CoV-2 variants) and subcellular (mutations in the spike surface of the virus, among others) levels, the virus circulating worldwide. At the same time, knowing the strains circulating in many human and non-human sources in different areas allowed us to deeply investigate if this pandemic started naturally or if the virus was yielded in the lab.

Despite the obvious advantages, many limitations to the implementation of forensic microbiology in legal contexts, especially with regard to knowledge of the human microbiome, still need to be overcome: most studies are small-scale; human microbiome show inter and intra-variability and temporal variations; the microbiome can be a mixture of different sources (suspect, victim, environment…); the variability of methods used (e.g., DNA control protocol, sequencing platform); the lack of standardization, standards, and guidelines that can be universally and legally applied; the lack of databases, genome references, and metadata; the lack of sufficient representative genetic and geographic coverage (e.g., most PMI studies are from the USA); and the limited access to human cadavers (most often animal models are used) in *postmortem* studies. Probably, one of the greatest obstacles that is common to general microbiology is the lack of a unique protocol with standard procedures both for DNA extraction and sequencing processes. Paradoxically, this lack of uniformity can also serve as an opportunity for fostering novel discoveries that might not have materialized if everyone adhered to the same rigid procedures [16]. In fact, the sequencing technology, itself, and computational resources can provide a technical bias hampering the accurate determination of microbial composition (454 technology, Illumina, Nanopore) [3,156].

With respect to the studies on *postmortem* microbial succession in particular, the vast majority of them are performed in animal models, as access to human cadavers is very limited. Thus, the translation of animal studies to the human scenario may be difficult to establish, making the interpretation of the results challenging [50]. Indeed, the most realistic approach to studying forensic microbiology during decomposition is to make use of human cadavers, but there are several reasons limiting their access and secured research. In addition, donated human cadavers can represent a highly variable initial microbiome, as already described. Different nonhuman animal models have been used as a proxy for humans in forensic decomposition (e.g., domesticated swine, rodents), especially in PMI estimation by forensic entomologists, as they are crucial to establishing significant patterns in microbial communities, taxonomy, and different variables under controlled lab settings. Some manipulation studies can even control, for example. the presence/absence of arthropods [157].

During the collection of samples for the calculation of PMI, the bacterial species isolated could have no cadaveric provenance due to sample contaminations. These contaminations can arise from different sources, including from non-sterile collection instruments or from the environment surrounding the cadaver. Therefore, because each sample presents specific traits, it is important to establish a contamination scale, which should be taken into consideration during data analysis [50]. The environmental variables that impact the accuracy of the models for PMI estimation remain largely unknown. Currently, the temperature represents the only environmental variable tested and considered by the models for PMI estimation; however, other variables, such as humidity, oxygen, and precipitation, greatly impact the bacterial profile overtime. Thus, it is of great importance to include such variables in the development of novel models for PMI calculation. In addition, it remains largely unknown which type of samples and which body location has the most accurate bacterial population for PMI estimation. All this knowledge will certainly help in developing novel, more robust, and accurate models for the estimation of the PMI [12,64]. A major limitation in the study of PMI using microbial succession is the fact that the most accurate period of the microbial clock remains unknown. It is considered that the microbial communities of decomposers should provide the most accurate estimative during periods of rapid microbial succession (early/active decay stage of decomposition) since bacteria have a large amount of nutrients available. However, large-scale data sets, with continuous sampling, for a long period of time, and taking into account temperature and humidity conditions, should be considered to validate such a hypothesis [64]. Overall, a large barrier to creating robust PMI models for the thanatomicrobiome knowledge is the lack of human cadaver-associated data sets from different environments, encompassing diverse populations and derived from standardized procedures.

## 7. Concluding Remarks and Future Perspectives

The crucial role that bacteria play in the human body decomposition process is now well established. They are present in various internal and external anatomic places of the body and derive not only from inside the corpse, but also from scavenging vertebrates, arthropods, and the soil where they are located, thus having a redoubled influence on the decomposition process. High-throughput sequencing technologies, complemented by robust bioinformatic workflows, have emerged as remarkable advancements in microbial analysis, namely by overcoming the challenges associated with identifying unknown, unculturable, and low-abundance microorganisms. Still, there is a clear need: (i) to develop standardized procedures for the collection, storage, analysis, and interpretation of microbiological evidence; (ii) to create complete and solid databases (including human metadata, such as geographic origin, ethnicity, diet, among others) which can be used in a forensic context, with microorganisms as complementary evidence in different criminal cases; (iii) to combine massive sequencing with complementary methods, such as metabolomics (e.g., through metabolomics it is possible to determine that microbial phosphate solubilization by *Pseudomonas* spp. plays a role in bone degradation); (iv) to perform studies with a much larger number of samples; and (v) to explore other microorganisms in addition to bacteria, for which most studies are dedicated. Finally, the increasing availability of genomic sequencing data and high-resolution microbial images provides a rich source of information for training Artificial Intelligence (AI) systems, which can identify valuable correlations within complex microbiological datasets and many variables towards more precise prognostic models [158]. AI can assume a pivotal role in formulating critical medicolegal conclusions in a range of forensic procedures, including the collection of samples of medicolegal significance from body cavities, the identification of pathological changes in various organs, or calculations related to the time since death. In the same way, it has the potential to improve our understanding of the complex interactions occurring in the human microbiome [159] and between the microbiome and human death [160].

The use of bacteria for forensic identification presents a compelling alternative to traditional methods, primarily due to their ease of sampling and the presence of a distinct microbial community for each individual. Given that the *postmortem* microbiota is primarily characterized by the dominance of Bacillota and Pseudomonadota, future research should focus on these key phyla to identify stable and reliable biomarkers that have the potential to revolutionize forensic sciences. Standardization of the methods employed in microbiological forensic analysis and their consolidation into a unified protocol is, however, needed to promote consistency, and comparability, and, ultimately, strengthen the position of microbiology in the fields of forensic sciences. Indeed, there is a bias along the sequencing process still in optimization/revolution and also in live microbiome studies. It is also crucial to establish the extent to which microbial community changes are influenced by various factors, such as environmental and soil conditions, and variations in the human microbiome and host species. Considering the huge variation of microbial communities affected by diverse intern and environmental factors, the development of a forensic microbial bank is urgently needed. In this aspect, the Human Postmortem Microbiome Project (HPMP) (https://hpmmdatabase.wixsite.com/hpmmdatabase/what-is-the-hpmm; last accessed on 30 June 2023), created in 2018, will provide robust datasets of *postmortem* microbial communities. regarding the abundance and diversity of microorganisms involved in human decomposition. In addition, it will surely advance death investigation by identifying microbial signatures or biomarkers for application in forensic sciences, including in PMI determinations, and by validating and standardizing protocols.

In the practical application of *postmortem* microbiology, forensic pathologists should take into account the following key considerations: (i) proper sampling techniques: the accuracy of results hinges on the use of appropriate sampling techniques; different clinical settings may demand distinct sampling protocols, so it is imperative to select the most suitable approach for each case; (ii) collaboration with microbiologists: to ensure the precise interpretation of results, forensic pathologists should establish collaborative relationships with microbiologists—their expertise is invaluable in navigating the complexities of microbiological findings; and (iii) interpretation of results: forensic pathologists must recognize the inherent limitations of *postmortem* microbiology; they should interpret the results with a keen awareness of these limitations and consider the broader context of the specific case at hand [161,162,163].

While forensic applications of the microbiome have benefited from well-established algorithms in both classification and regression tasks, such as k-nearest neighbors, random forest models, and neural networks, machine learning methods offer clear advantages in handling complex and multidimensional microbiome data, but quantitative computation of relevant forensic parameters is required. The ability to refine error rates related to PMI estimates and other forensic applications from the microbiome knowledge will further determine if microorganisms can be effectively used in judicial processes. Such approaches align perfectly with the famous Locard’s principle “every contact leaves a trace” who realized that physical evidence would be left at virtually every crime scene [164]. Only robust and comprehensive investigations, potentially supported by machine learning models, can bridge these knowledge gaps toward a reliable approach to the use of microorganisms in judicial contexts.

## Data Availability

No new data were created.
